# Substance Use and Depression Symptomatology: Measurement Invariance of the Beck Depression Inventory (BDI-II) among Non-Users and Frequent-Users of Alcohol, Nicotine and Cannabis

**DOI:** 10.1371/journal.pone.0152118

**Published:** 2016-04-05

**Authors:** Ashlee A. Moore, Michael C. Neale, Judy L. Silberg, Brad Verhulst

**Affiliations:** 1 Center for Clinical and Translational Research, Virginia Commonwealth University, Richmond, VA, United States of America; 2 Virginia Institute for Psychiatric & Behavioral Genetics, Virginia Commonwealth University, Richmond, VA, United States of America; 3 Department of Human and Molecular Genetics, Virginia Commonwealth University, Richmond, VA, United States of America; 4 Department of Psychiatry, Virginia Commonwealth University, Richmond, VA, United States of America; Universidad de Granada, SPAIN

## Abstract

Depression is a highly heterogeneous condition, and identifying how symptoms present in various groups may greatly increase our understanding of its etiology. Importantly, Major Depressive Disorder is strongly linked with Substance Use Disorders, which may ameliorate or exacerbate specific depression symptoms. It is therefore quite plausible that depression may present with different symptom profiles depending on an individual’s substance use status. Given these observations, it is important to examine the underlying construct of depression in groups of substance users compared to non-users. In this study we use a non-clinical sample to examine the measurement structure of the Beck Depression Inventory (BDI-II) in non-users and frequent-users of various substances. Specifically, measurement invariance was examined across those who do vs. do not use alcohol, nicotine, and cannabis. Results indicate strict factorial invariance across non-users and frequent-users of alcohol and cannabis, and metric invariance across non-users and frequent-users of nicotine. This implies that the factor structure of the BDI-II is similar across all substance use groups

## Introduction

There is a longstanding debate regarding whether depression is a homogeneous disorder, or whether it is a collection of inter-related disorders [1,2]. Depressed mood is generally thought of as the prototypical depressive symptom [3], but variations in the presentation of depressive symptoms are often reported. For example, decreased anhedonia and increased somatization are common in older adults with depression [4,5], whereas irritability is often a primary component of depression in adolescents and young adults [3,5,6]. In addition, an “externalizing” or “aggressive” type of depression has been proposed for some males experiencing depression [7,8]. This debate has been further exacerbated by the notable lack of replicated molecular genetic findings for Major Depressive Disorder [9], despite significant heritability estimates from twin studies [10]. If depression is a heterogeneous condition, identifying symptom presentation in various groups may greatly increase our understanding of its etiology.

Importantly, Major Depressive Disorder (MDD) is strongly associated with Substance Use Disorders (SUDs) [11,12,13,14]. One potential reason for this association is the self-medication hypothesis [15,16], whereby individuals use substances in attempt to control or treat their depression. If substance use ameliorates or exacerbates specific symptoms, then levels of depression symptoms may differ depending on an individual’s substance use profile. Furthermore, by acting on specific symptoms, substance use may reduce their diagnostic validity, such that their presence or absence would be random. The specific symptom that was “treated,” however, would be a function of the type of substances used. In such cases the frequency with which the symptom is reported would not necessarily differ between individuals with different substance use profiles, but the item may become less relevant to the underlying construct of depression.

While there are several methods of assessing depression, the Beck Depression Inventory (BDI-II) [17] is one of the most frequently used self-report measures, assessing depressive symptoms across the spectrum from not depressed to severely depressed. Furthermore, the BDI-II can be self-administered without clinical supervision, and has been shown to have good psychometric properties, including convergent validity [18,19], internal consistency [17,18,19] and test-retest reliability [17]. However, little research has examined the psychometric properties of the BDI-II among persons with varying levels of substance use, especially among non-clinical or sub-clinical populations.

The psychometric approach known as measurement invariance [20] provides a method to test whether the properties of a measurement instrument vary across groups. Formally, measurement invariance is “the mathematical equality of corresponding measurement parameters for a given factorially defined construct (i.e., the loadings and intercepts of a construct’s multiple manifest indicators) across two or more groups” [21] (p 55). Simply put, tests of measurement invariance examine whether an instrument’s items operate the same way in different groups. If there *is* measurement invariance of the BDI-II, there are *no differences* in the measurement, function, or meaning of its items as a function of substance use. Alternatively, if there is a *failure* of measurement invariance of the BDI-II with respect to substance use status, then at least one of the items functions differently in substance users relative to non-users. If a difference is found, the next step is to identify *which* items function differently. Importantly, if a failure of measurement invariance is ignored, then comparing means between groups is confounded by the fact that different constructs are being measured.

Measurement Invariance can be statistically investigated by fitting a multiple group common factor model [20]. In this method, confirmatory factor analysis (CFA) is used to estimate parameters that reflect differences in the latent construct that the items are intended to assess. The primary parameters of interest include factor loadings (λ), item means (μ) and item residuals (δ). Factor loadings indicate change of each item for a 1-unit increase in the latent factor, with higher scores indicating stronger relationships. Item means indicate the average value for each item, and item residuals indicate the variance for each item after accounting for the latent construct. These parameters are jointly considered in the examination of measurement invariance.

**Fig 1:**
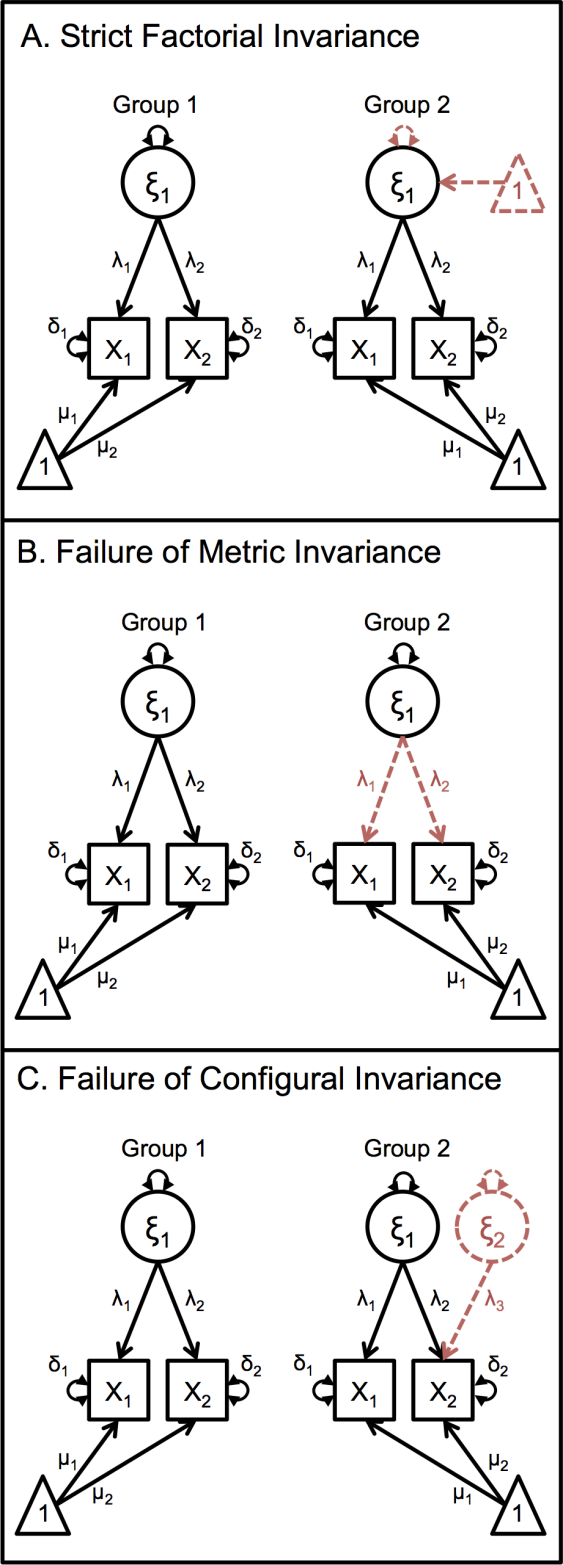
Types of Measurement Invariance. Types of measurement invariance, with parameters unable to be equated displayed in red and dashed. (A) Strict factorial invariance, with between group differences arising from different factor means and/or different factor variances. (B) Failure of metric invariance, with between group differences arising from different factor loadings. (C) Failure of configural invariance, with between group differences arising from different numbers of latent factors.

The measurement invariance status of a set of items is usually classified into one of four types. First is strict factorial invariance (Fig 1A), in which the number of factors is the same across groups and factor loadings, item means and variances are not significantly different. Under strict factorial invariance, the mean(s) and variance(s) of the latent factor may be different, but the other measurement components are equivalent, implying that the same construct is being measured in all groups. In this case, mean differences between the groups are solely a function of differences in the latent factor, and represent true, interpretable differences. For example, one group may have higher levels of depression, or more variance in depression, but the underlying factor structure (i.e., factor loadings and residuals) is the same. Note that differences in factor mean(s) and variance(s) can only be interpreted if the other parameters can be equated. Thus, investigators should ensure that these parameters are equal before concluding that mean or variance differences exist (or don’t) between groups. If a researcher ignores measurement non-invariance, they may be comparing proverbial apples and oranges, rather than how many apples each group has.

The first type of measurement non-invariance, failure of metric invariance (Fig 1B), involves differences in factor loadings across groups. An example of this is the study by Aggen and colleagues [22] which found that fatigue is more characteristic of depression in older adults, whereas feelings of worthlessness are less characteristic of depression in older females (age x sex effect). Accordingly, comparing mean levels of depression in groups where there is a failure of metric invariance is problematic, as the characteristic depression symptomatology of each group is different.

The second type of measurement non-invariance, failure of configural invariance (Fig 1C), occurs when the number of latent factors differs across groups, or when the items that load on the latent factors in one group are not the same in the other group. In an extreme case, it is possible for one group to have a three-factor solution, and the other group to have a one-factor solution. Comparison of the three-factor solution to the one-factor solution would typically be impossible. An exception might occur if one of the factors in the three-factor group was invariant to the factor in the single-factor group, but such an occurrence is likely quite rare. In failures of both metric and configural invariance, the underlying constructs differ, making group mean or variance comparisons prone to error.

### Measurement Invariance of the BDI-II in Substance Use Groups

To date, five studies have focused on identifying the factor structure of the BDI-II among substance use groups [23,24,25,26,27]. In the four that examined the factor structure of the BDI-II in substance users, a three-factor model consisting of cognitive, affective, and somatic constructs appears to fit the data best [23,25,26,27]. In addition, when comparing people with and without alcohol abuse, Skule et al. [24] found this same three-factor model was the best fitting solution for both groups.

We are aware of two studies that have examined measurement invariance of the BDI-II among those with and without SUDs; both found measurement invariance [23,24]. In a recent study of 525 clinically depressed individuals, Skule and colleagues [24] found support for measurement invariance of the BDI-II across those with and without alcohol abuse. Seignourel, Green & Schmitz [23] also found support for measurement invariance of the BDI-II across substance dependence classes (alcohol, opioid and cocaine). However, to our knowledge, no study to date has examined measurement invariance in sub-clinical substance users, nor in nicotine or cannabis use groups.

### Study Aims / Hypotheses

The aims of the current study were two-fold. First, as the existing literature suggests that substance abusers have higher levels of depression, our first aim was to replicate this finding in a non-clinical substance use sample by assessing mean differences in depression symptomatology across non-users and frequent-users of alcohol, nicotine and cannabis. Second, we sought to assess the measurement structure of the BDI-II across these groups to determine if the BDI-II has the same fundamental meaning for each group. While support for measurement invariance of the BDI-II has been found across alcohol abusers and non-abusers [24], no one has yet examined this topic in non-clinical alcohol use groups, nor in any sample involving nicotine or cannabis use. Thus, our second aim was to replicate the finding of measurement invariance of the BDI-II in a non-clinical alcohol use sample, as well as explore the possibility of supporting measurement invariance in nicotine and cannabis use groups.

Hypothesis A (H_A_): BDI-II item response means differ significantly between non-users and frequent-users of each substance. Null hypothesis A (H0_A_): BDI-II item response means do not differ significantly between groups.

Hypothesis B (H_B_): BDI-II factor loadings do not differ significantly between non-users and frequent-users of each substance (measurement invariance). Null hypothesis B (H0_B_): BDI-II factor loadings differ significantly between groups (measurement non-invariance).

## Materials and Methods

### Participants

Participants were recruited through Amazon’s Mechanical Turk website (MTurk.com) as part of a larger study on substance use and psychopathology. Previous research has shown MTurk to be a reliable method of data collection, with Cronbach alphas and test-retest reliability meeting or exceeding other methods of data collection [28]. In total, 603 United States residents completed the study, with a mean age of 33.3 years (range = 18.5–71.2). The majority of participants were female (54.6%) and Caucasian (78.3%). The institutional review board of Virginia Commonwealth University provided ethical approval for this research, and written informed consent was obtained from all participants.

### Measures

Participants were administered extended questionnaires to assess a variety of traits. To decrease participant burden and maximize the breadth of the study, a planned missing data strategy was used, in which participants responded to a random set of questionnaire items. Accordingly, not all participants answered all questions, but data are missing completely at random and therefore the parameter estimates are unbiased [29]. Only participants who completed both the BDI-II and substance use items (*N* = 282) are included in the current analyses.

#### Beck Depression Inventory, Second Edition (BDI-II)

The BDI-II [17] is a self-report measure of depression consisting of 21 items. Each item is rated on a 4-point Likert scale, with higher scores representing greater severity of depression. In the current study, the item assessing suicidal thoughts was not administered to participants due to concerns by the IRB about the assessment of suicidality in an online survey, resulting in a total of 20 administered BDI-II items. S1 Table provides a list of response options for each BDI-II item assessed in the current study.

#### Substance Use Measures

In order to capture reasonably frequent substance use, which does not necessarily mean abuse or dependence, we compared substance non-users to frequent-users. Due to the non-clinical nature of our sample, as well as the high percentage of substance non-users (approximately 37% for alcohol, 77% for nicotine and 70% for cannabis), we chose cut-offs for frequent substance use that would maximize group differences while maintaining reasonable sample sizes. Substance use categorizations were made based on self-report, which is the most widely used method to assess substance use, and is generally regarded as a valid measurement technique for these phenotypes [30,31,32]. Table 1 displays sample characteristics stratified by substance use category. Specific categorizations for each substance are presented below.

**Table 1:** Sociodemographic characteristics and Beck Depression Inventory means for non-users and frequent-users of alcohol, nicotine and cannabis. Total and stratified by substance use group.

	Alcohol		Nicotine		Cannabis		Total
	Non-Users N (%)	Frequent-Users N (%)	Non-Users N (%)	Frequent-Users N (%)	Non-Users N (%)	Frequent-Users N (%)	N (%)
**Total N**	105 (37.2)	85 (30.1)	218 (77.3)	49 (17.4)	196 (69.5)	62 (22.0)	282 (100.0)
**Gender**							
Male	45 (42.9)	46 (54.1)	111 (51.4)	17 (34.7)	83 (42.6)	34 (55.7)	134 (47.9)
Female	60 (57.1)	39 (45.9)	105 (48.6)	32 (65.3)	112 (57.4)	27 (44.3)	146 (52.1)
**Race**							
White	84 (80.0)	74 (87.0)	176 (81.1)	45 (91.8)	159 (81.1)	51 (83.6)	228 (81.1)
Black	10 (9.5)	5 (5.9)	20 (9.2)	2 (4.1)	17 (8.7)	4 (6.6)	23 (8.2)
Asian	5 (4.8)	4 (4.7)	7 (3.2)	2 (4.1)	9 (4.6)	3 (4.9)	13 (4.6)
Hispanic	4 (3.8)	2 (2.4)	9 (4.1)	0 (0.0)	8 (4.1)	2 (3.3)	12 (4.3)
Other	2 (1.9)	0 (0.0)	5 (2.3)	0 (0.0)	3 (1.5)	1 (1.6)	5 (1.8)
**Age**							
Age [Mean (SD)]	33.9 (11.9)	32.6 (9.8)	32.4 (10.4)	36.1 (11.1)	33.6 (10.9)	31.6 (9.9)	33.0 (10.5)
**BDI-II**							
Score [Mean (SD)]	29.5 (10.6)	29.5 (11.2)	28.9 (10.0)	30.9 (11.8)	29.2 (10.5)	29.7 (11.2)	29.5 (10.6)

Participants were assigned to one of two alcohol use groups based on their responses to two questions: “have you ever had an alcoholic drink?” and “how many drinks do you have in a typical week?” If participants indicated that they had ever consumed an alcohol drink, then they were asked to indicate how many drinks they consume in a typical week: 0 drinks, 1 to 3 drinks, 4 to 6 drinks, 7 to 12 drinks, 13 to 18 drinks, 19 to 24 drinks, 25 to 42 drinks, or more than 42 drinks. Participants who endorsed either never having consumed an alcoholic drink, or consuming 0 drinks in a typical week were assigned to the alcohol non-user group (N = 105, 37.2% of the sample). Frequent alcohol users were defined as participants who consumed 4 or more drinks in a typical week (N = 85, 30.1% of the sample). Occasional drinkers, or participants who endorsed consuming between 1 and 3 drinks a week were excluded from the analyses. S1 and S2 Figs display the BDI-II item distributions for alcohol non-users and frequent-users, respectively.

Respondents were assigned to one of two nicotine use groups based on their responses to two questions. In the first question, participants were asked “what category best describes your tobacco use history?” Response options included “never smoked or used smokeless tobacco,” “smoked or used smokeless tobacco on and off,” “use to use tobacco but quit,” and “currently smoke or use smokeless tobacco.” Among those who endorsed currently using tobacco, a second question was asked: “how frequently did you smoke cigarettes in the last 30 days?” Response options included 0 days, 1 to 2 days, 3 to 4 days, 5 to 11 days, 12 to 14 days, 15 to 25 days, and 26 to 30 days. Participants who endorsed either never using tobacco, having quit using tobacco previously, or smoking 0 cigarettes in the last 30 days were assigned to the nicotine non-user group (N = 218, 77.3% of the sample). Participants who endorsed smoking cigarettes 15 or more days during the last 30 days were assigned to the nicotine frequent-user group (N = 49, 17.4% of the sample). Very few participants (less than 6%) endorsed smoking between 1 and 14 cigarettes in the last 30 days, and these individuals were excluded from the analyses. S3 & S4 Figs display the BDI-II item distributions for nicotine non-users and frequent-users, respectively.

Respondents were assigned to one of two cannabis use groups based on their response to two questions: “on how many occasions (if any) have you used marijuana (weed, pot, cannabis) or hashish (hash, hash oil) in your lifetime?” and “on how many occasions (if any) have you used marijuana (weed, pot, cannabis) or hashish (hash, hash oil) in the last 12 months?” Only participants who endorsed using cannabis in their lifetime were asked about their past 12-month use. Response options for both questions were 0 times, 1 to 2 times, 3 to 5 times, 6 to 9 times, 10 to 19 times, 20 to 39 times, and 40 or more times. Participants who endorsed either never using cannabis, or using cannabis 0 times in the last 12 months were assigned to the cannabis non-user group (N = 196, 69.5% of the sample). Participants who endorsed using cannabis 3 or more times in the last 12 months were assigned to the cannabis frequent-user group (N = 62, 22% of the sample). Those who endorsed using cannabis 1 to 2 times in the previous 12 months were excluded from the analyses. S5 & S6 Figs display the BDI-II item distributions for cannabis non-users and frequent-users, respectively.

### Statistical Analysis

To determine the number of BDI-II factors that would be retained for each substance use group, a polychoric correlation matrix was estimated between the ordinal items, and the eigenvalues of this matrix were computed. Parallel analysis [33] using 1,000 permutations of the ordinal data was examined and the 97.5% eigenvalue quantile threshold was determined. Original eigenvalues that exceeded this threshold were retained in subsequent analyses. For each group, only a single factor exceeded the parallel analysis threshold, and thus only a single factor was retained for each group. S7 Fig presents the eigenvalue scree plots [34] for each group, with Kaiser rule [35] threshold (eigenvalue > 1) displayed in red, and parallel analysis [33] threshold displayed in blue.

To test for differences in depression symptom levels across groups, we examined a series of difference of means tests for ordinal variables, in which the first two thresholds were constrained to zero and one, respectively, and the third threshold was freely estimated for each group. These constraints allow for the estimation of the mean and variances of the items, assuming the liability-threshold model, making these tests analogous to t-tests for continuous variables [36].

To test for measurement invariance in the BDI-II we examined a series of three nested structural equation models for each substance use group comparison (alcohol, nicotine and cannabis). As with the difference of means tests, we used a threshold model in order to account for the ordinal nature of the BDI-II items. Because there was evidence for a single depression factor across non-users and frequent-users of each substance (configural invariance), Model 1 served as our base model by specifying the single-factor model and allowing factor loadings to be freely estimated for each group. Model 2 examined failure of metric invariance by equating factor loadings across groups. Model 3 assessed strict factorial invariance by equating all parameters and then freeing the factor means and factor variance. Likelihood ratio tests were used to examine model fit and determine the best fitting model for each substance. A significant deterioration in fit for any of these models indicates a failure of measurement invariance at that respective level. These procedures were repeated for all three substances (alcohol, nicotine and cannabis), comparing non-users to frequent-users.

Parallel analyses and ordinal difference of means tests were performed in OpenMx 2.0 [37] using the R software package [38]. Measurement invariance models were performed in MPlus version 6.11 [39].

## Results

First, we tested differential levels of BDI-II item-level depression symptomatology by running a series of ordinal difference of means tests. Following Mehta, Neale & Flay [36], in these tests the first two thresholds were constrained to zero and one, respectively, and the third threshold was freely estimated for the non-users and frequent-users for each substance (alcohol, nicotine & cannabis). These threshold constraints permit estimation of the mean and variance of the underlying item dimension. Table 2 presents the χ^2^ statistic and p-value for each BDI-II item for the ordinal difference of means tests.

**Table 2:** Likelihood ratio tests (χ^2^ and p-value) for ordinal difference of means tests for Beck Depression Inventory items between non-users and frequent-users of alcohol, nicotine and cannabis.^*^

Item	Alcohol		Nicotine		Cannabis	
	χ^2^	p	χ^2^	p	χ^2^	p
1	< 0.001	.984	1.303	.254	0.549	.459
2	0.678	.410	0.208	.649	0.253	.615
3	0.127	.722	1.637	.201	**6.366**	**.012**
4	0.343	.558	2.493	.114	1.852	.174
5	0.459	.498	**7.767**	**.005**	0.700	.403
6	0.384	.536	3.234	.072	0.284	.594
7	3.496	.062	0.325	.569	2.910	.088
8	0.299	.584	0.728	.393	0.417	.519
9	2.032	.154	0.365	.545	0.099	.753
10	0.970	.325	1.056	.304	0.240	.624
11	2.517	.113	2.321	.128	< 0.001	.996
12	0.301	.583	1.515	.218	1.011	.315
13	0.223	.637	0.663	.415	0.316	.574
14	0.108	.743	0.060	.807	0.008	.927
15	0.009	.925	0.189	.664	3.314	.069
16	0.200	.655	0.107	.743	0.345	.557
17	0.823	.364	**4.175**	**.041**	0.626	.429
18	0.039	.843	0.683	.409	0.211	.646
19	1.903	.168	2.016	.156	< 0.001	1.00
20	2.016	.156	0.181	.671	7.706	.054

* Bold indicates a significant difference of means test at α = .05

For alcohol non-users and frequent-users, there were no significant differences in the reported means of item-level BDI-II depression symptomatology. For nicotine, feelings of guilt (item 5) and changes in appetite (item 17) were significantly higher in the frequent-user group than in the non-user group. For cannabis, feelings of failure (item 3) were significantly higher in the frequent-user group than in the non-user group. Overall, the average level of BDI-II depression item endorsement appears quite similar for non-users and frequent-users of alcohol, nicotine, and cannabis. Although three items did show significant differences across groups, none of these differences would remain significant after Bonferroni multiple testing correction. Thus, H0_A_ was not clearly rejected for any of the three substances.

Table 3 presents the goodness-of-fit statistics for the measurement invariance models tested in each substance category. For these models, a significant difference in likelihood ratio tests indicates a significant deterioration in model fit, and thus suggests a failure of measurement invariance. For alcohol and cannabis, the strict factorial invariance model did not significantly deteriorate model fit, indicating that all model parameters could be equated across non-users and frequent-users of both alcohol and cannabis. In other words, the measurement structure for the BDI-II does not appear to be significantly different across non-users and frequent-users of alcohol and cannabis. For nicotine, the metric invariance model did not significantly deteriorate model fit, indicating that the factor loadings could be equated across non-users and frequent-users of nicotine. However, the significant deterioration in fit for the nicotine strict factorial invariance model indicates that item thresholds were unable to be equated across nicotine use groups. In other words, the factor structure of the BDI-II does not appear significantly different across non-users and frequent-users of nicotine, but the proportion of people in each response category is significantly different (analogous to different item means and residual variances for continuous variables). Thus, we failed to reject H0_B_ for all three substances. Table 4 presents the factor loading estimates for non-users and frequent-users of each substance under the factorial invariance models.

**Table 3:** Measurement invariance model goodness-of-fit statistics for non-users and frequent-users of alcohol, nicotine and cannabis.

	Model	Estimated Parameters	CFI	RMSEA	χ^2^	Δdf	p
Alcohol non-users vs. frequent-users	Configural Invariance	140	0.985	0.042	-	-	-
	Metric Invariance	120	0.997	0.017	17.242	20	0.6372
	Factorial Invariance	82	0.988	0.035	57.661	58	0.4879
Nicotine non-users vs. frequent-users	Configural Invariance	140	0.991	0.030	-	-	-
	Metric Invariance	120	0.992	0.028	26.385	20	0.1535
	Factorial Invariance	82	0.985	0.037	88.655	58	0.0059
Cannabis non-users vs. frequent-users	Configural Invariance	138	0.981	0.047	-	-	-
	Metric Invariance	118	0.994	0.026	15.218	20	0.7638
	Factorial Invariance	78	0.994	0.023	52.745	60	0.7355

**Table 4:** Factor loading (λ) estimates for Beck Depression Inventory items for non-users and frequent-users of alcohol, nicotine, and cannabis, under the factorial invariance model.

Parameter	Alcohol non-users and frequent users	Nicotine non-users and frequent users	Cannabis non-users and frequent users
	Estimate (SE)	Estimate (SE)	Estimate (SE)
λ_1_	0.810 (.046)	0.772 (.046)	0.781 (.037)
λ_2_	0.661 (.058)	0.713 (.050)	0.705 (.043)
λ_3_	0.761 (.047)	0.765 (.050)	0.773 (.036)
λ_4_	0.782 (.045)	0.777 (.047)	0.760 (.034)
λ_5_	0.837 (.043)	0.829 (.037)	0.795 (.033)
λ_6_	0.810 (.043)	0.850 (.037)	0.774 (.035)
λ_7_	0.726 (.052)	0.743 (.050)	0.746 (.038)
λ_8_	0.844 (.036)	0.879 (.038)	0.825 (.027)
λ_9_	0.784 (.046)	0.767 (.051)	0.740 (.041)
λ_10_	0.830 (.041)	0.801 (.047)	0.816 (.032)
λ_11_	0.870 (.036)	0.832 (.041)	0.781 (.038)
λ_12_	0.755 (.048)	0.761 (.046)	0.770 (.034)
λ_13_	0.707 (.047)	0.794 (.044)	0.769 (.036)
λ_14_	0.809 (.042)	0.800 (.047)	0.788 (.037)
λ_15_	0.853 (.043)	0.819 (.043)	0.788 (.037)
λ_16_	0.848 (.036)	0.798 (.047)	0.800 (.035)
λ_17_	0.760 (.043)	0.806 (.043)	0.759 (.041)
λ_18_	0.812 (.042)	0.830 (.040)	0.826 (.033)
λ_19_	0.847 (.041)	0.857 (.041)	0.810 (.034)
λ_20_	0.811 (.038)	0.809 (.042)	0.797 (.029)

## Discussion

Our results indicate that a single-factor structure fit the data of the current sample best, with all items loading onto a single latent construct. Item-level depression symptomatology, as measured by the BDI-II, did not differ significantly across non-users and frequent-users of alcohol, nicotine or cannabis. Moreover, the measurement structure of the BDI-II is not significantly different for non-users and frequent users of alcohol, nicotine and cannabis.

Given that previous research on the factor and measurement structure of the BDI-II has generally used clinically ascertained samples [23,24,25] our study provides an important extension to the depression literature by examining the BDI-II structure in a non-clinical sample using sub-clinical depression and substance use measures. Specifically, we were able to replicate the previous the findings of measurement invariance in clinical alcohol use samples in a non-clinical, sub-threshold sample. Our study also extends the limited research on the BDI-II in users of nicotine and cannabis by examining the measurement structure of the BDI-II in users of these substances for the first time.

The current results should be interpreted in light of four potential limitations. First, we were unable to include the BDI-II item that assesses suicidal ideation, which could display significant differences across substance use groups. Second, study participants were not provided guidelines for reporting alcohol consumption (e.g., portion size and alcohol content), which could complicate the measurement of alcohol consumption in the current study. Third, our sample sizes were relatively small (between n = 49 and n = 218 per group), making it more difficult to reject null hypotheses. Finally, due to the use of a single sample, a poly-substance user could be included in all three analyses as a frequent-user. Conversely, a total abstainer from all three substances would be included as a non-user in all analyses. In an ideal scenario the samples would be independent, making it impossible for an individual to be in the control group (or frequent-user group) for more than one substance use category. However, inspection of the data revealed that the frequency of poly-users (defined as frequent-users of all three substances) and total abstainers (defined as non-users for all three substances) was relatively low (n = 13 and n = 71; ~ 5% and ~ 25% of the total sample, respectively). Accordingly, the majority of the sample (~70%) was a non-user of one substance and a frequent-user of another substance.

The current results, especially the nicotine and cannabis use analyses, require replication before firm conclusions can be drawn. Replication with larger samples, as well as using other measures of depression is encouraged.

## Conclusions

Our results provide further support for the use of the BDI-II, specifically among substance-using populations. The BDI-II appears to measure the same construct in both non-users and frequent-users of alcohol, nicotine and cannabis. Interestingly, no item level mean differences in depression symptomatology were found between non-users and frequent-users of any substance, suggesting that symptom-specific levels of depression are approximately equal among substance use groups. We encourage future research on mean level differences in depression symptomatology in sub-clinical substance-users.

## Supporting Information

S1 Fig BDI-II Item Distributions for Alcohol Non-users. BDI-II item-level distributions for alcohol non-users (N = 105).
(PDF)

S2 Fig BDI-II Item Distributions for Alcohol Frequent-users. BDI-II item-level distributions for alcohol frequent-users (N = 85).
(PDF)

S3 Fig BDI-II Item Distributions for Nicotine Non-users. BDI-II item-level distributions for nicotine non-users (N = 218).
(PDF)

S4 Fig BDI-II Item Distributions for Nicotine Frequent-users. BDI-II item-level distributions for nicotine frequent-users (N = 49).
(PDF)

S5 Fig BDI-II Item Distributions for Cannabis Non-users. BDI-II item-level distributions for cannabis non-users (N = 196).
(PDF)

S6 Fig BDI-II Item Distributions for Cannabis Frequent-users. BDI-II item-level distributions for cannabis frequent-users (N = 62).
(PDF)

S7 Fig Results of Exploratory Factor Analyses. Results of exploratory factor analysis for each substance use group. Scree plots are displayed plotting the eigenvalues extracted for each group: A) alcohol non-users, B) alcohol frequent-users, C) nicotine non-users, D) nicotine frequent-users, E) cannabis non-users, F) cannabis frequent-users. Kaiser rule threshold (eigenvalue > 1) is displayed in red, and results of 1,000 parallel analyses displayed in blue. Eigenvalues above the parallel analysis threshold were retained.
(TIFF)

S1 Table BDI-II Item Response Options. (DOCX)

S1 File Raw Data. (CSV)
