# Supplementary figures and images for: Substance Use and Depression Symptomatology: Measurement Invariance of the Beck Depression Inventory (BDI-II) among Non-Users and Frequent-Users of Alcohol, Nicotine and Cannabis
Source: PLoS One. 2016 Apr 5;11(4):e0152118. doi: 10.1371/journal.pone.0152118 (PMC4821457; doi:10.1371/journal.pone.0152118)

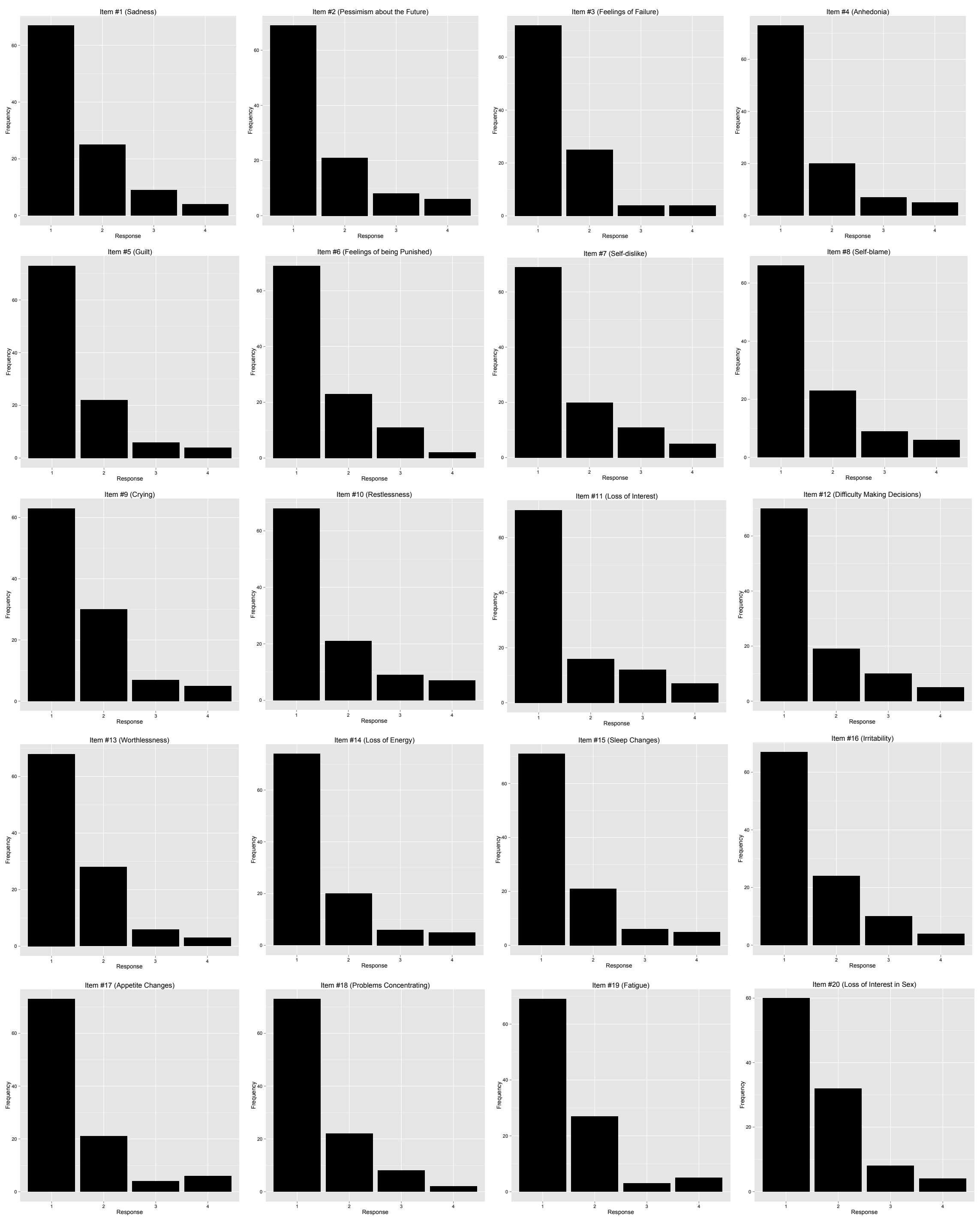

Supplement: S1 Fig — BDI-II item-level distributions for alcohol non-users (N = 105). (PDF) [file pone.0152118.s001.pdf]

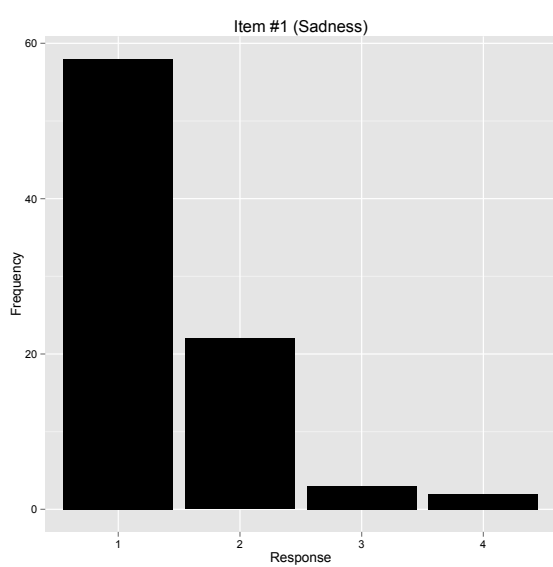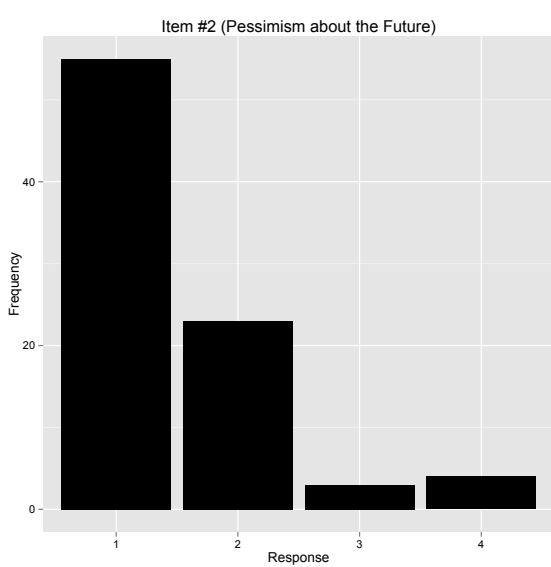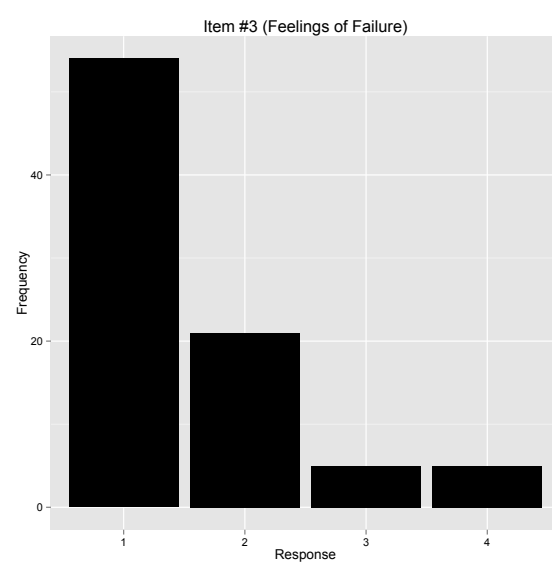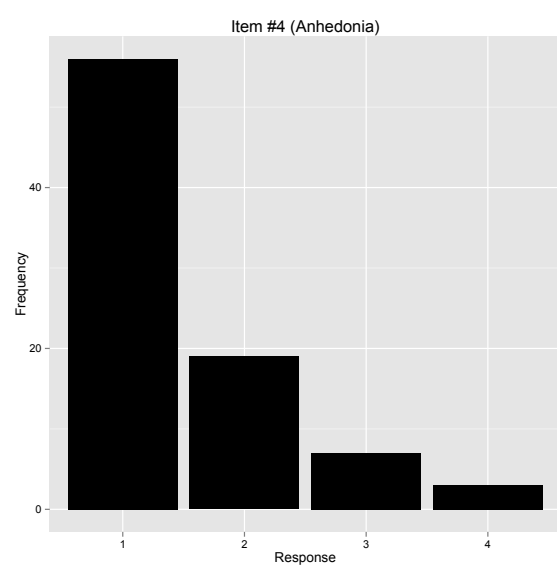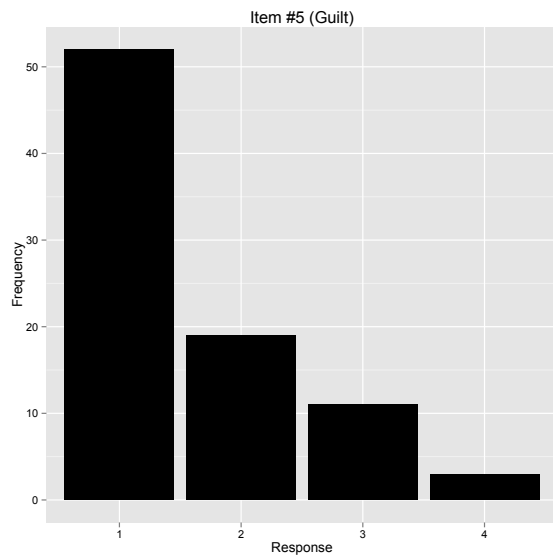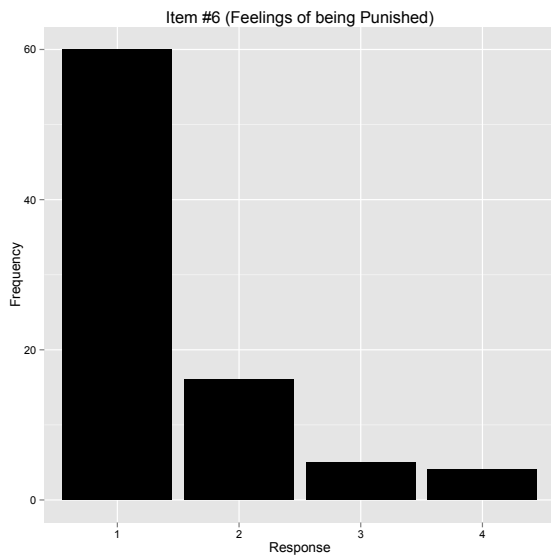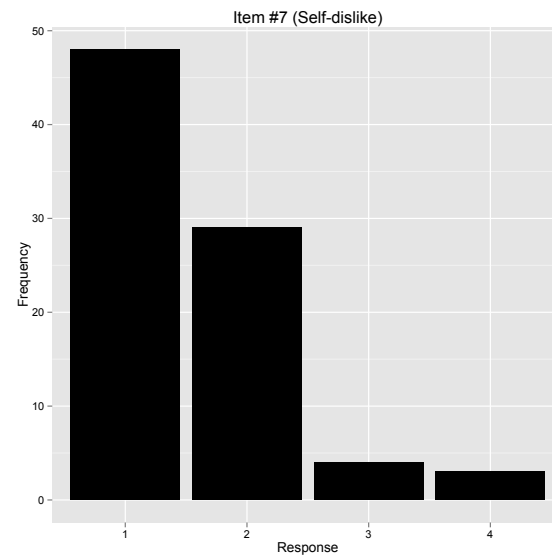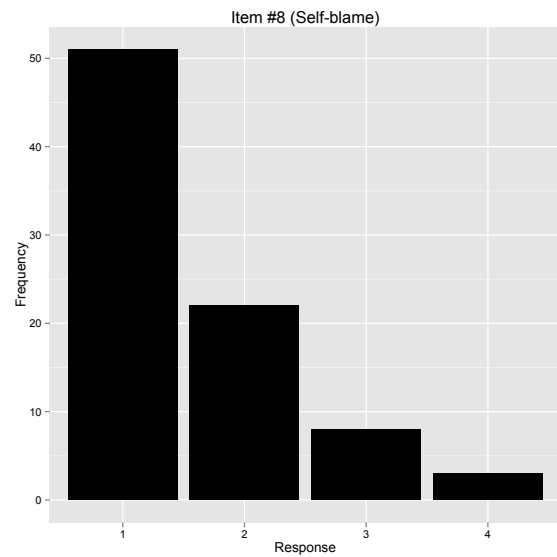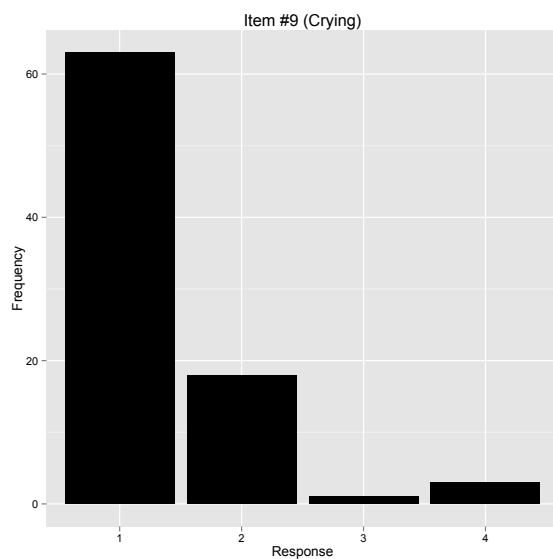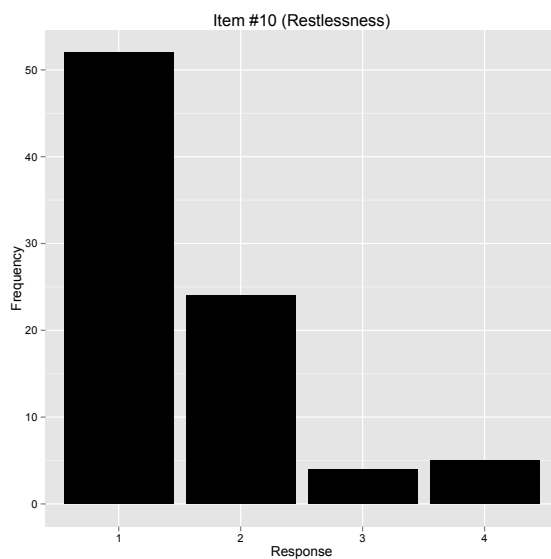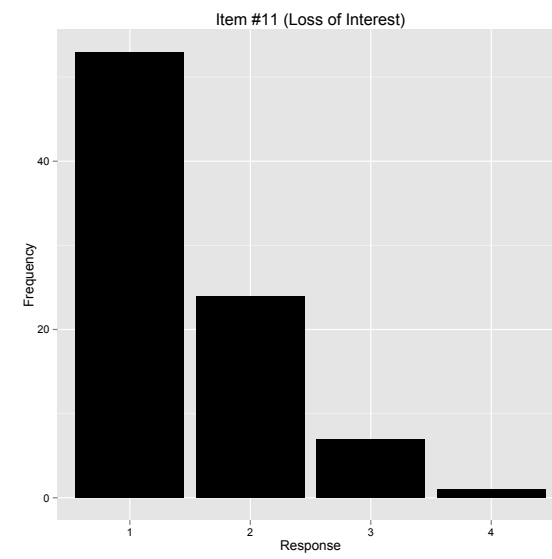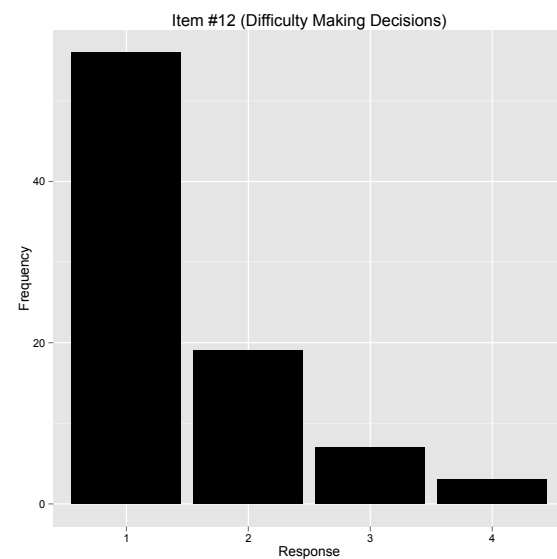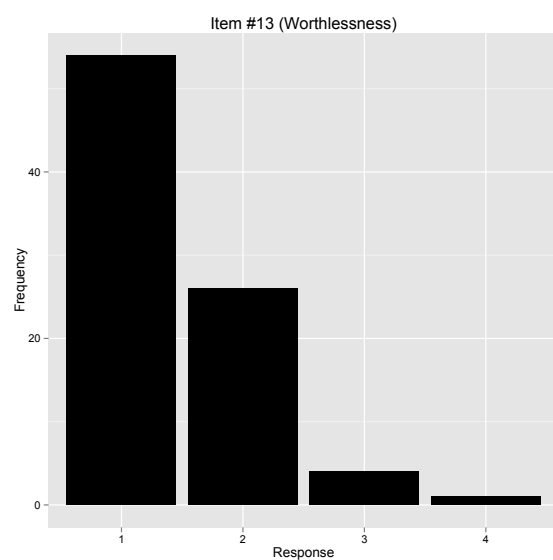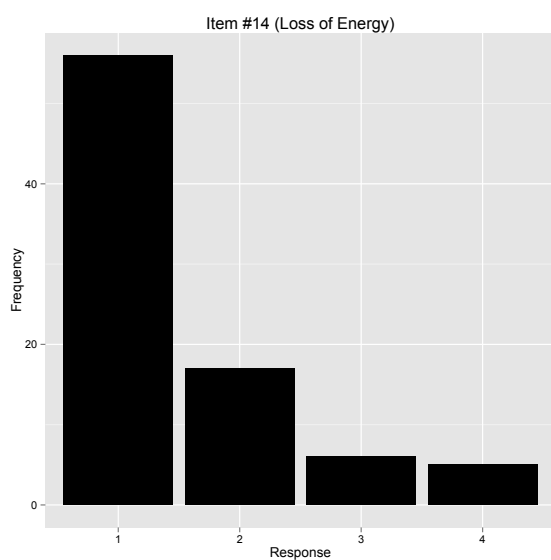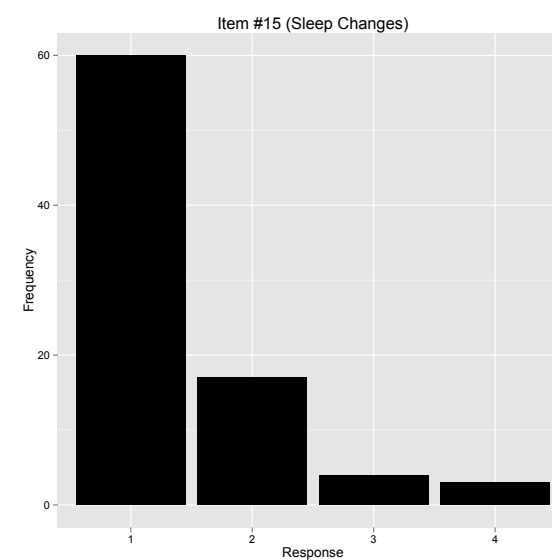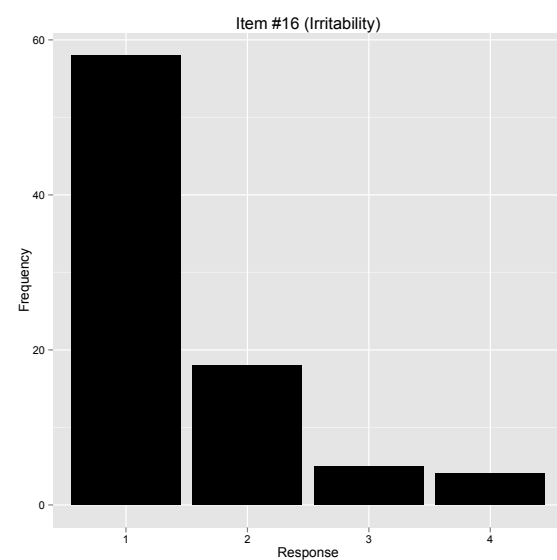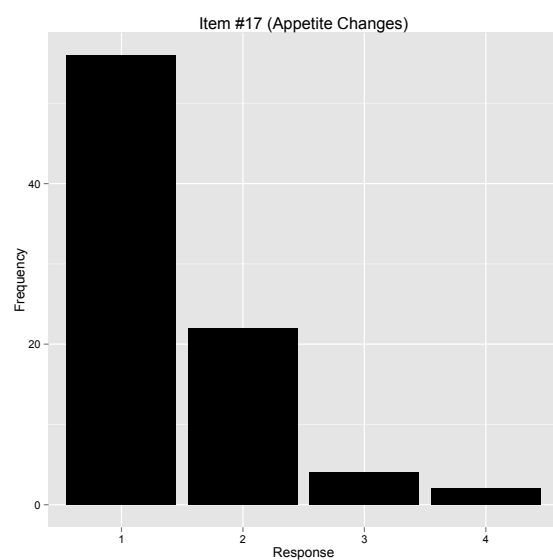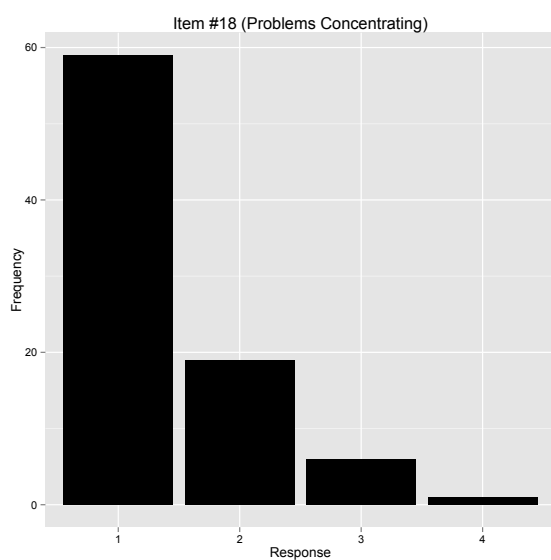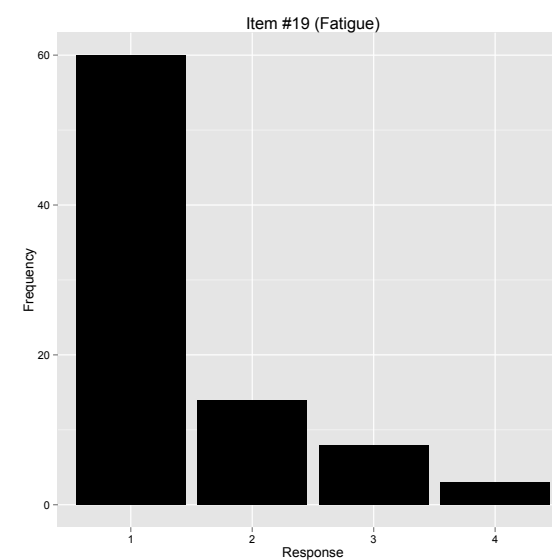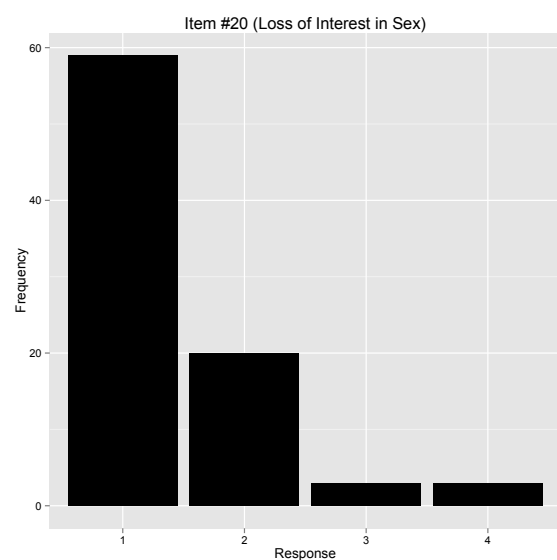

Supplement: S2 Fig — BDI-II item-level distributions for alcohol frequent-users (N = 85). (PDF) [file pone.0152118.s002.pdf]

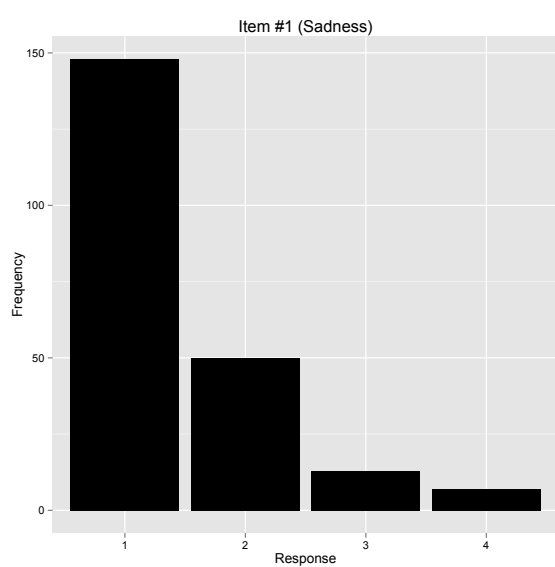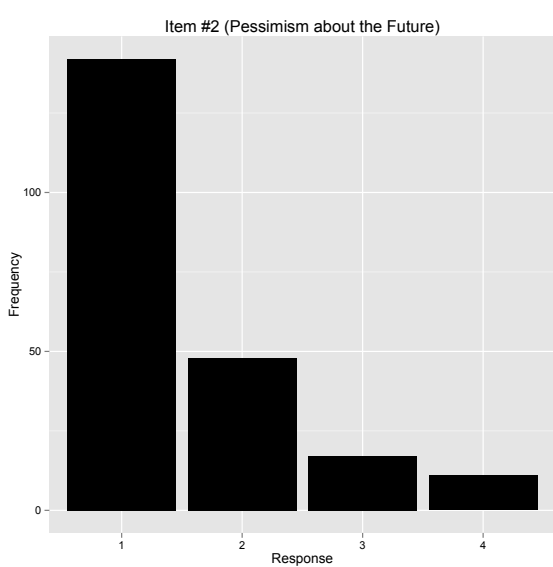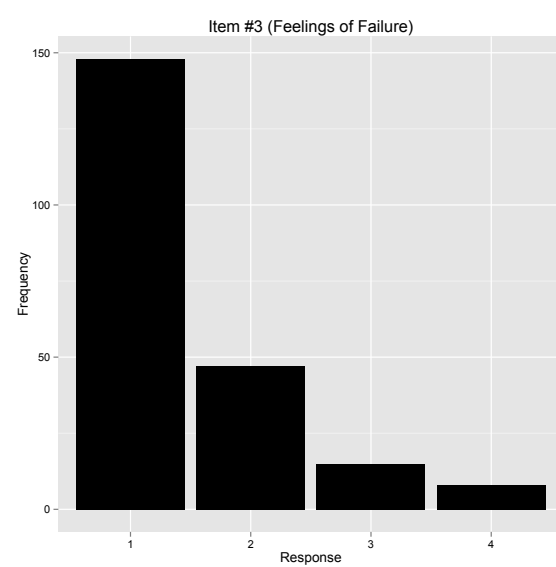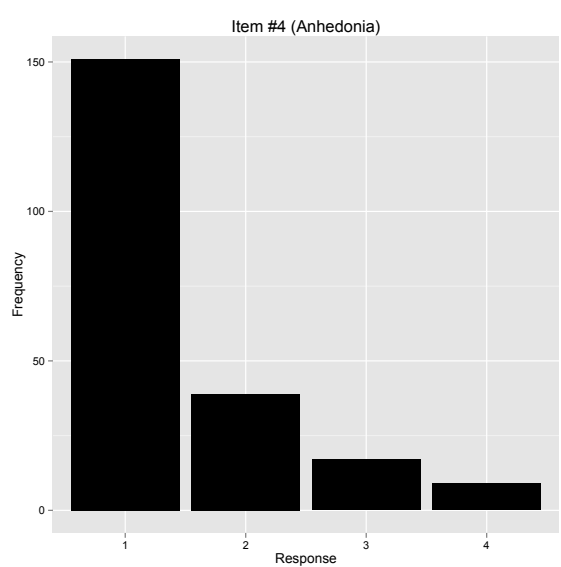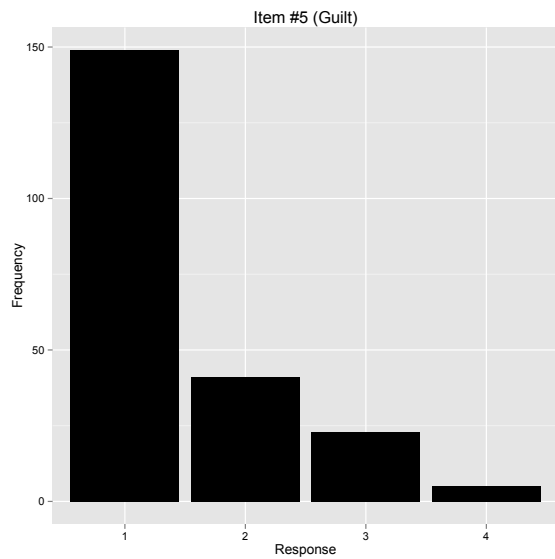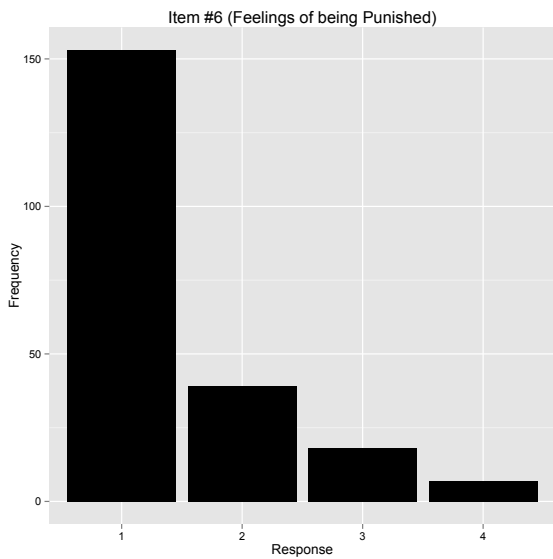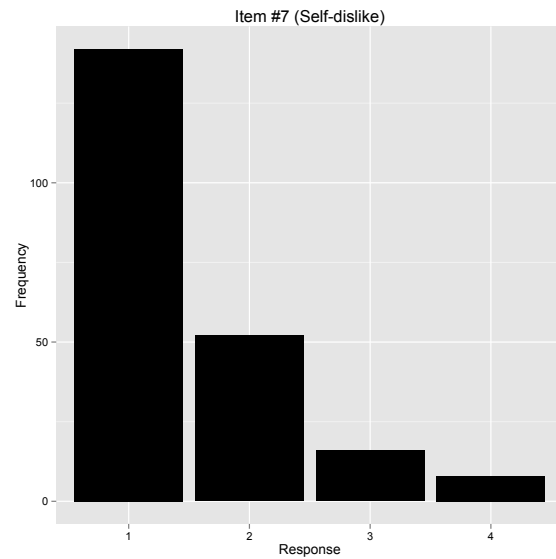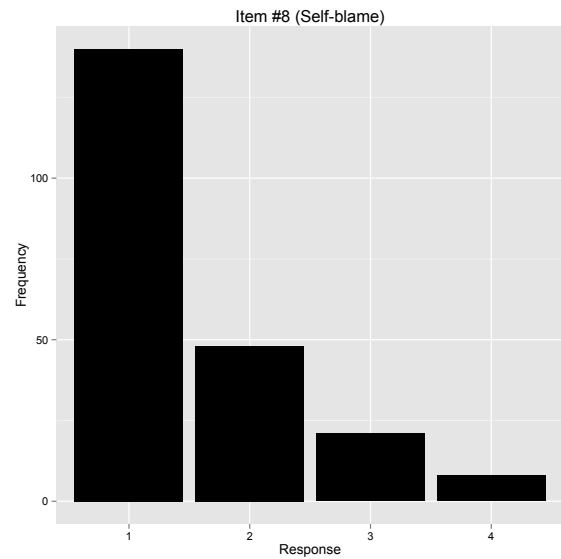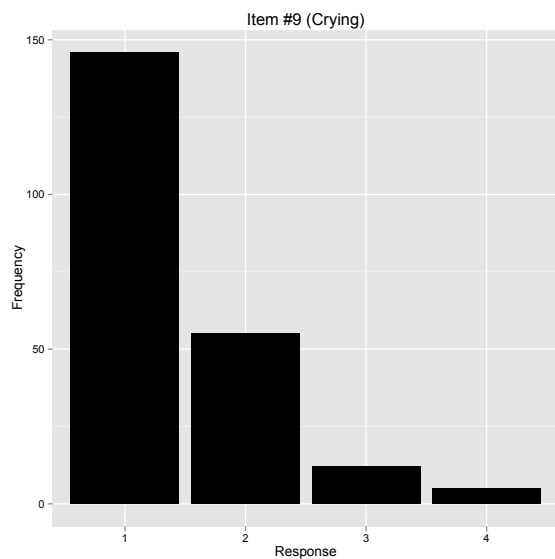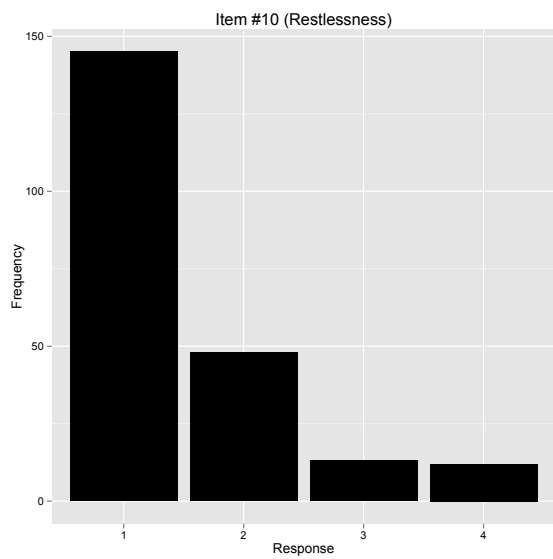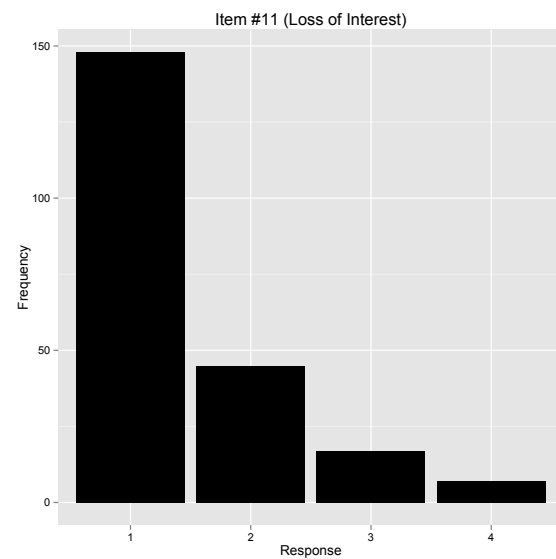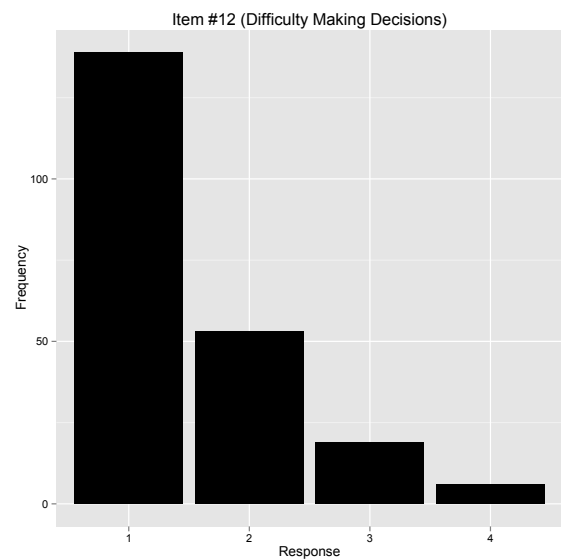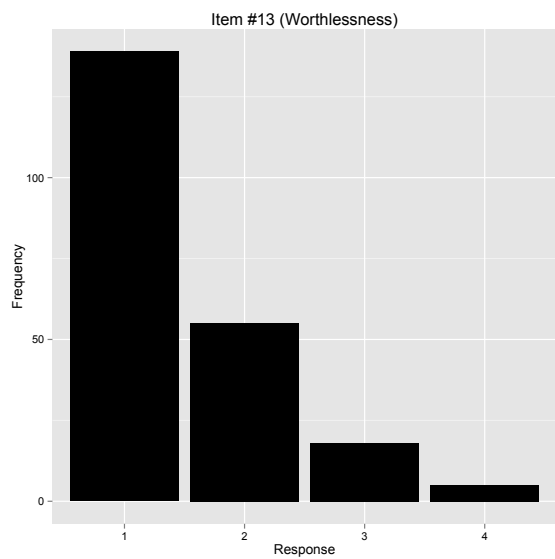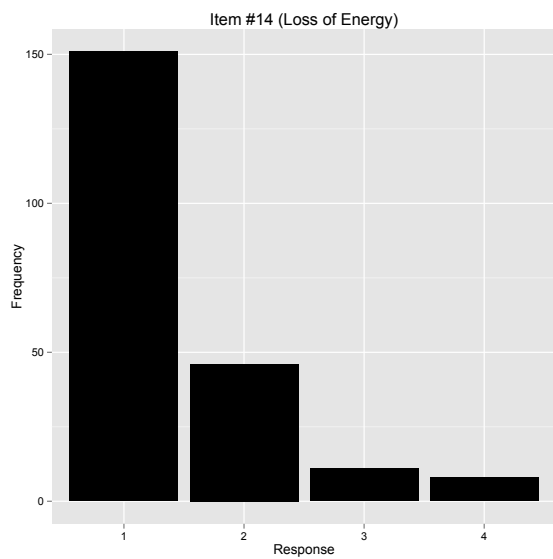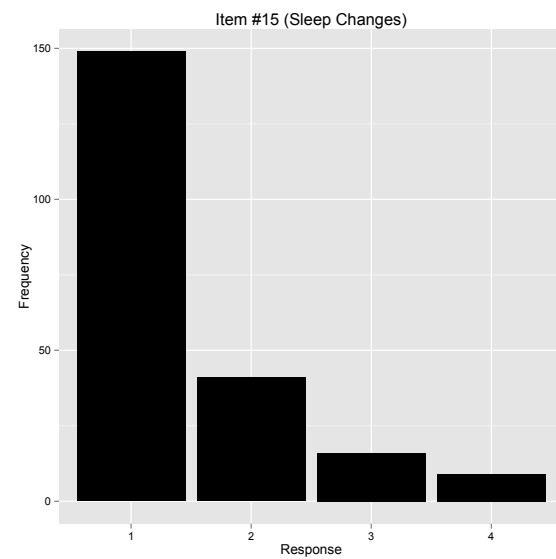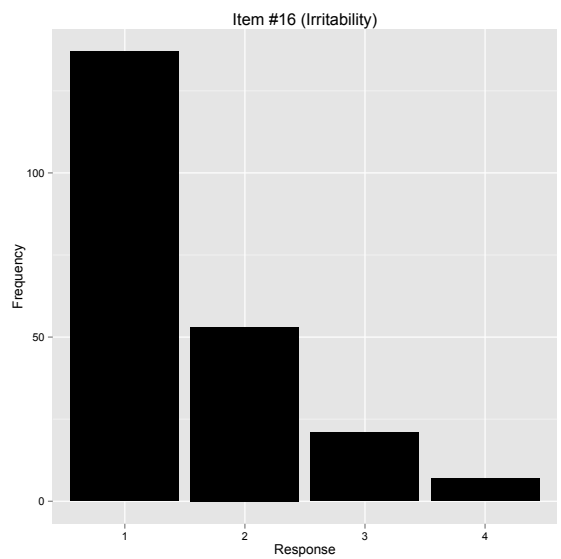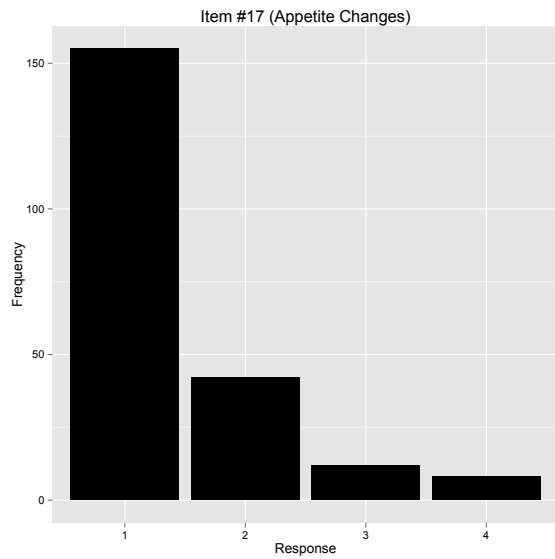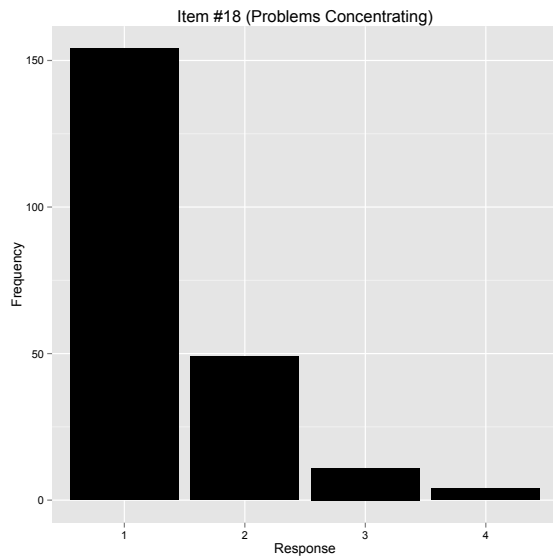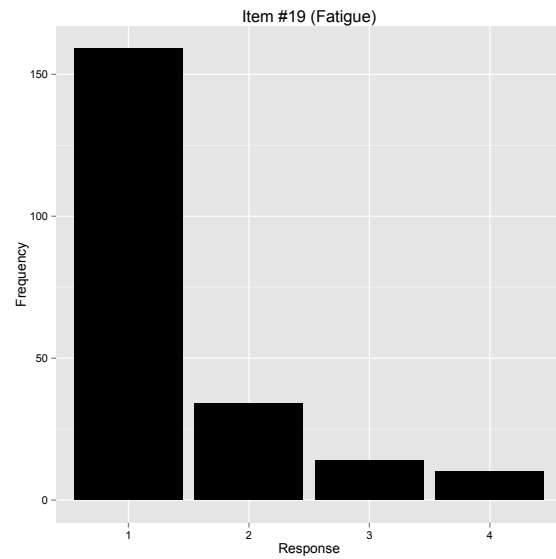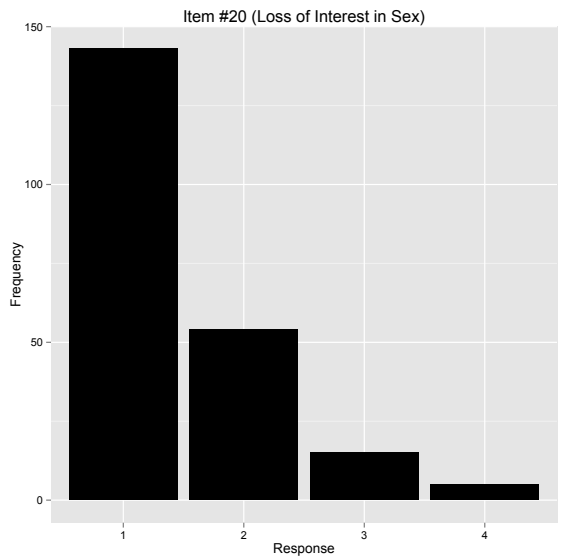

Supplement: S3 Fig — BDI-II item-level distributions for nicotine non-users (N = 218). (PDF) [file pone.0152118.s003.pdf]

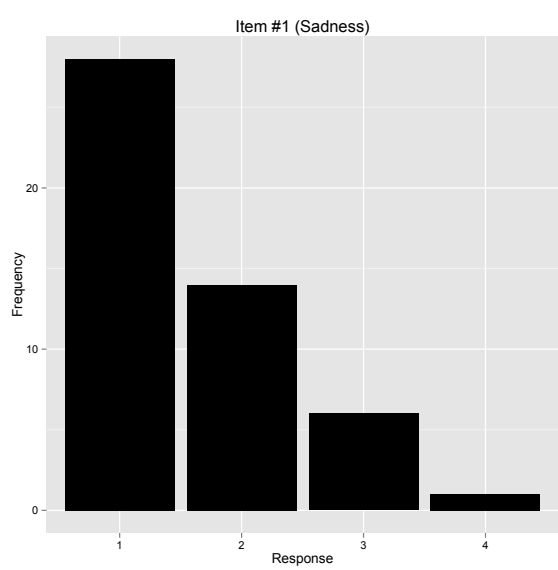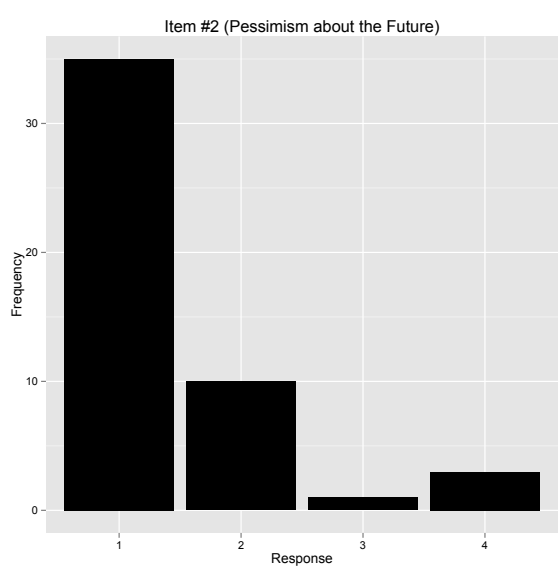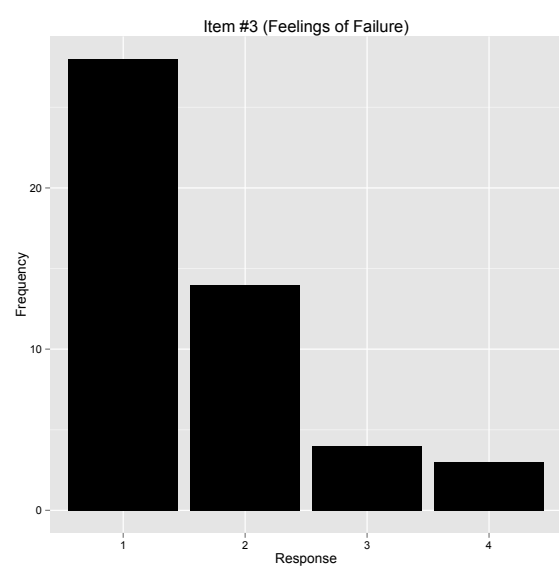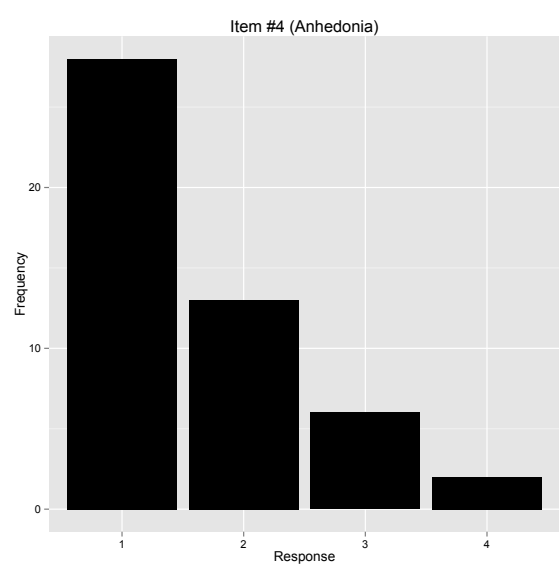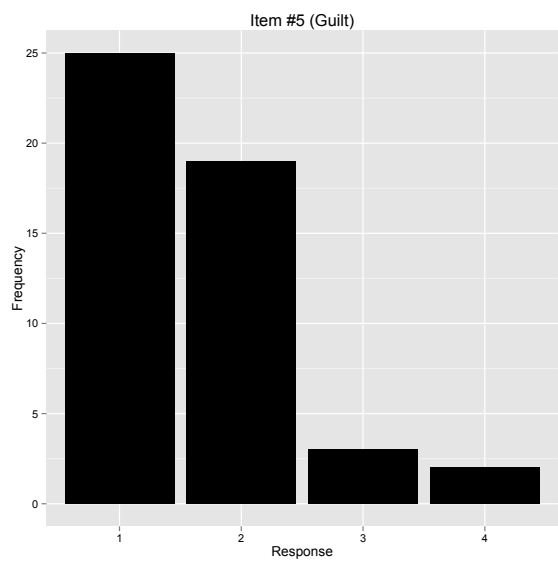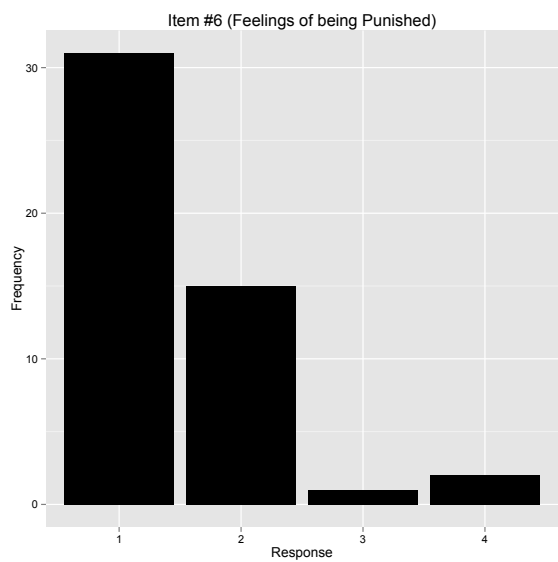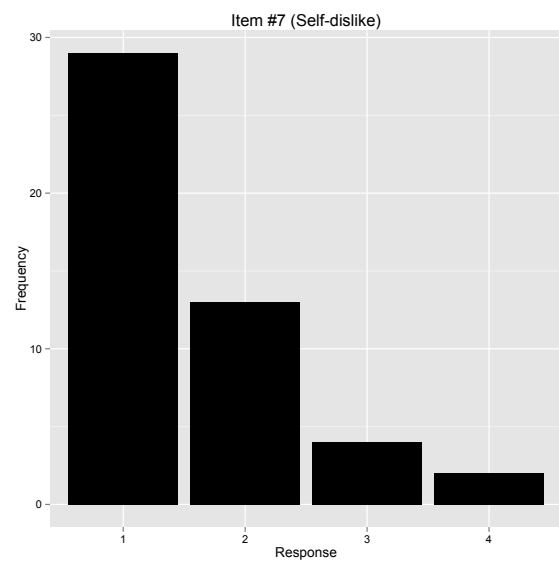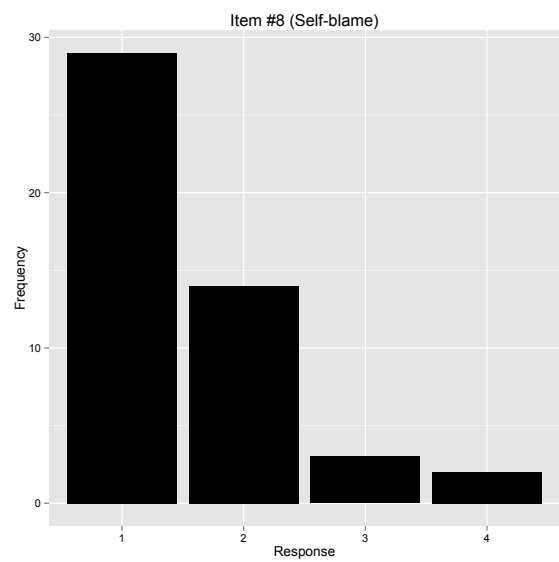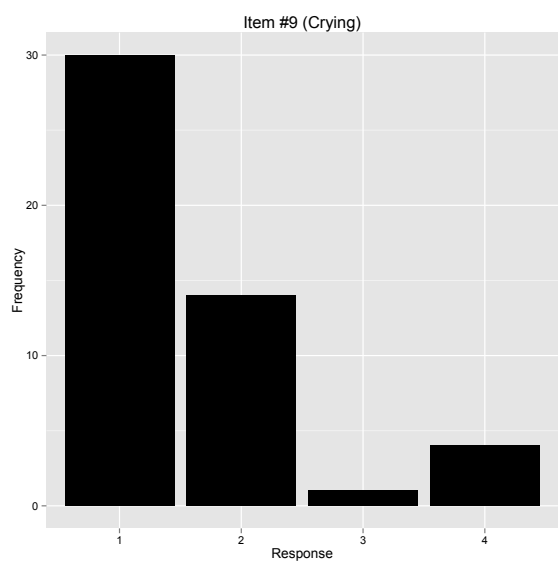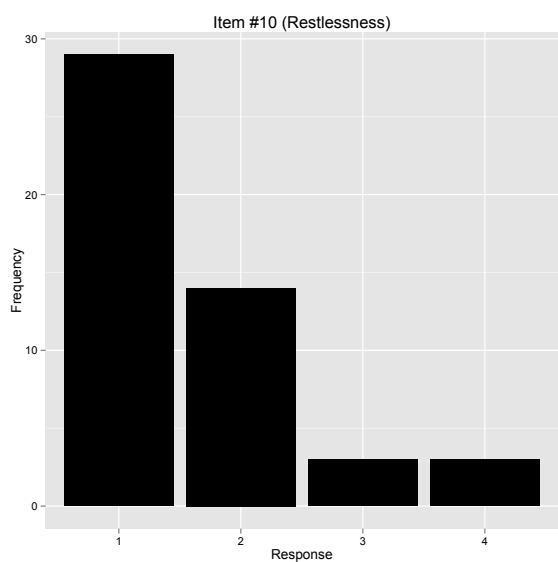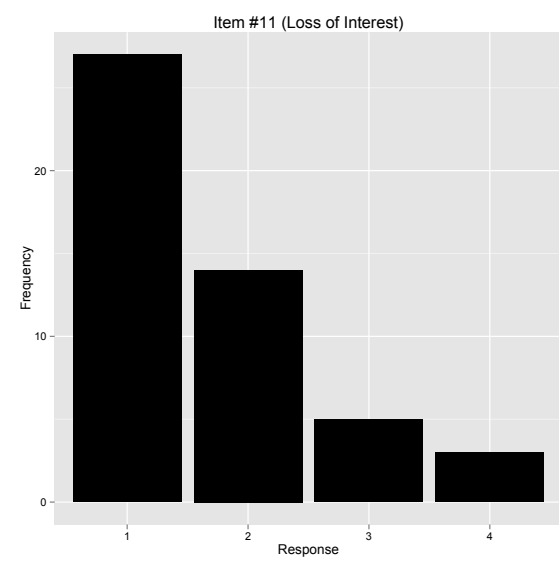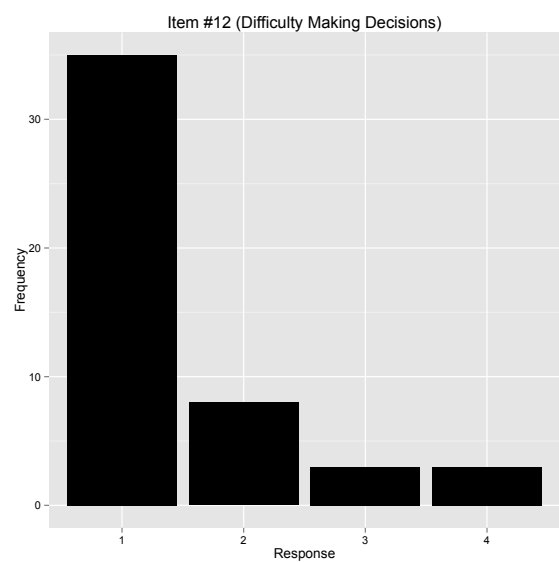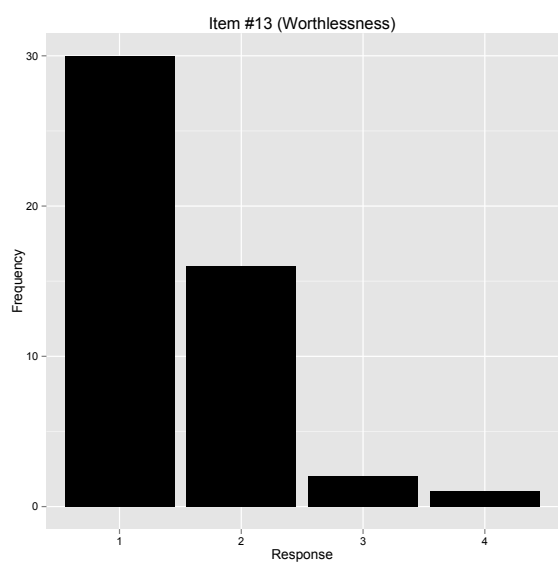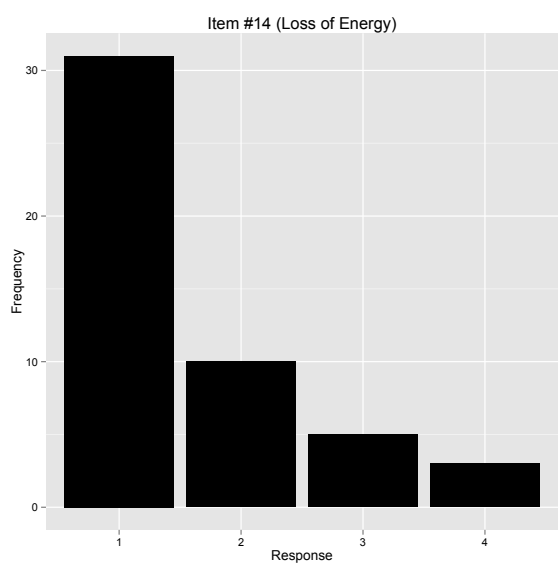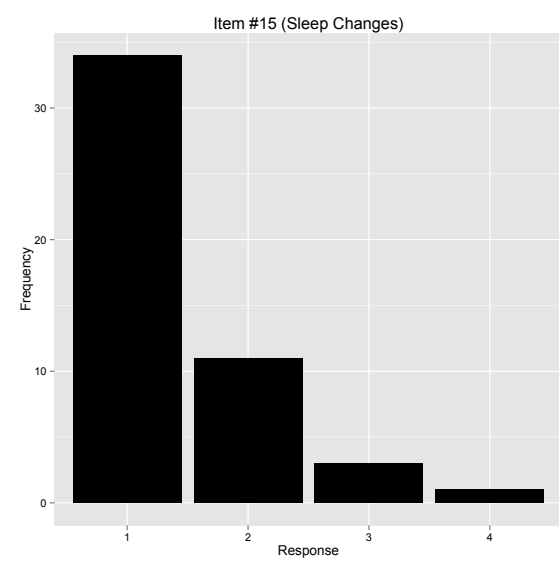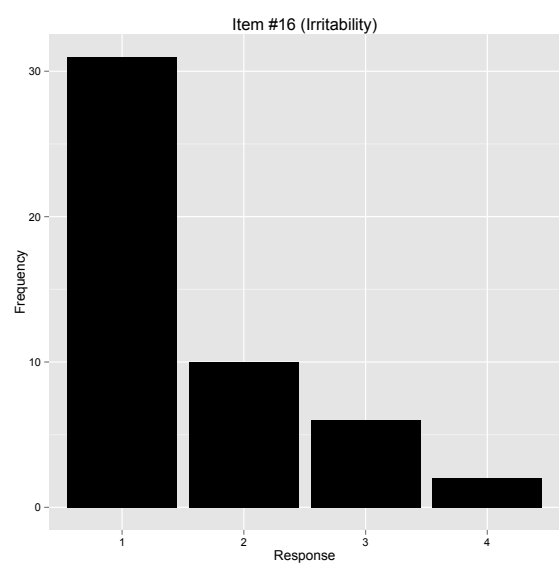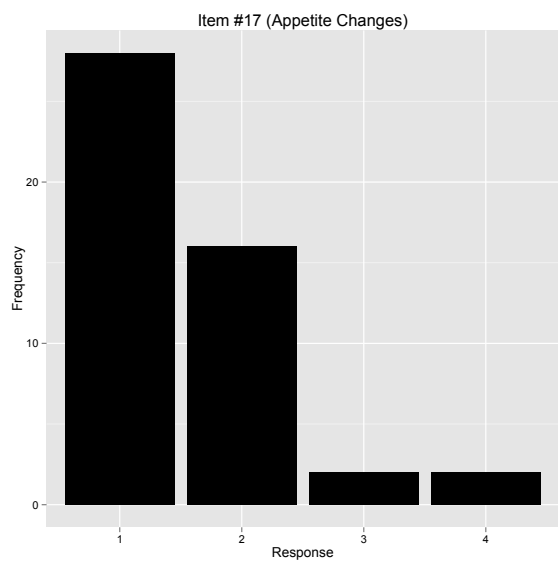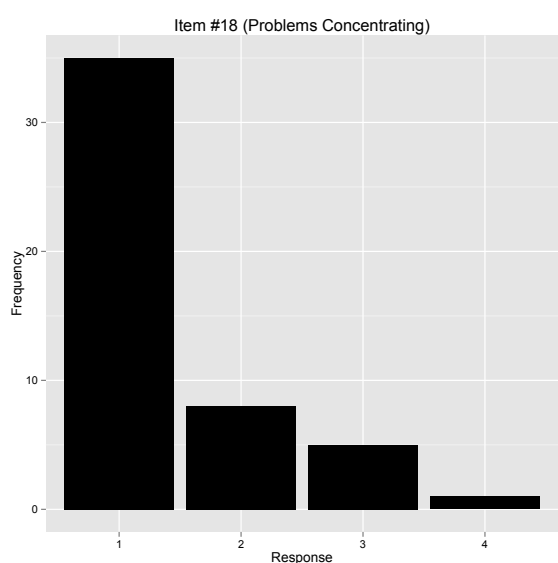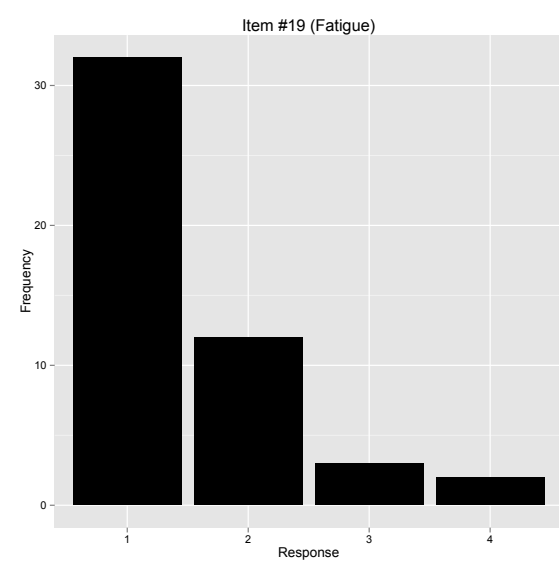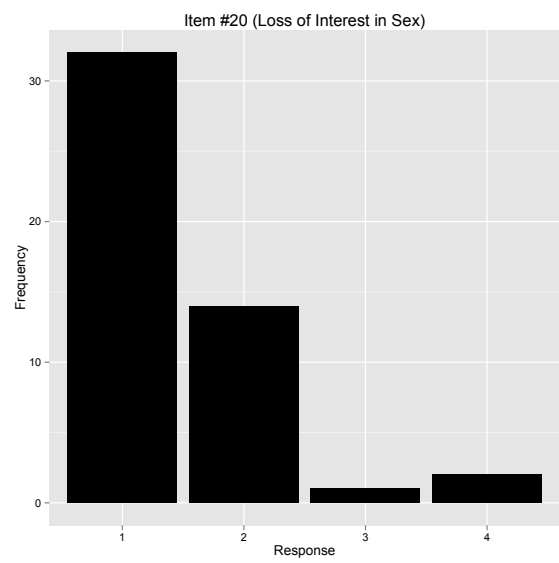

Supplement: S4 Fig — BDI-II item-level distributions for nicotine frequent-users (N = 49). (PDF) [file pone.0152118.s004.pdf]

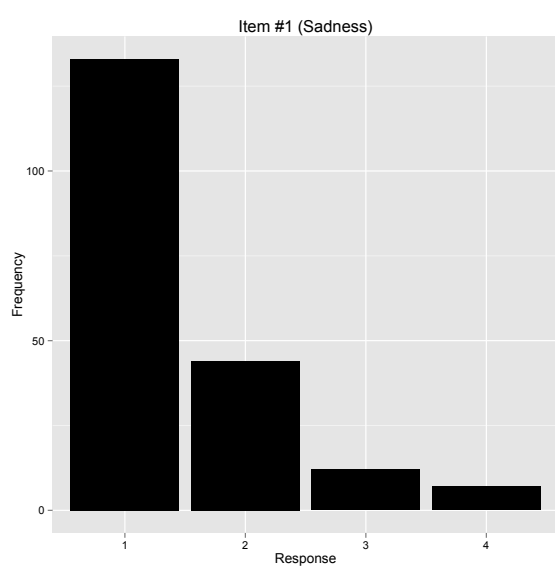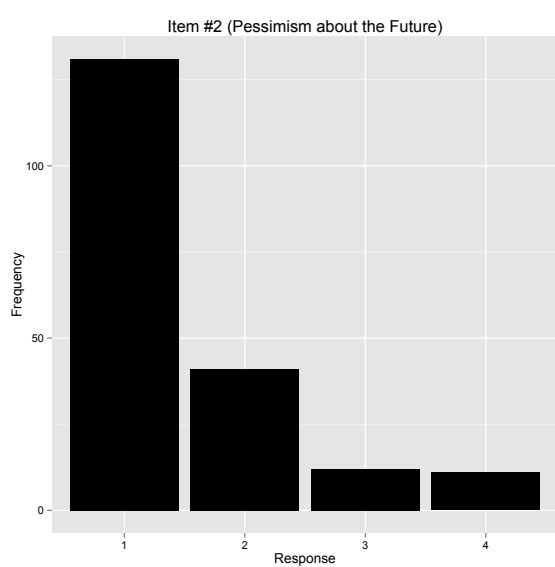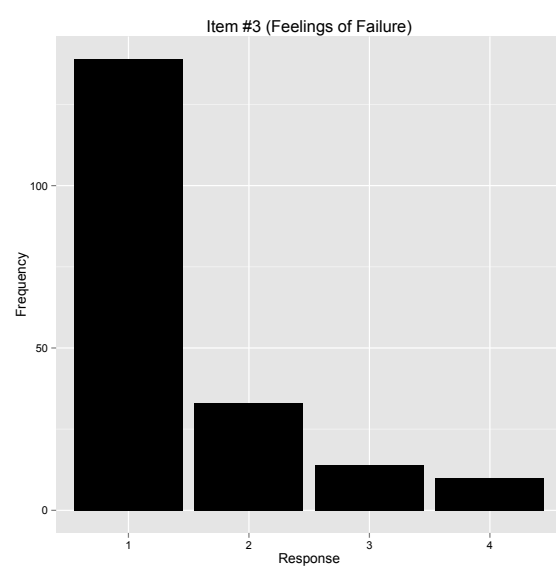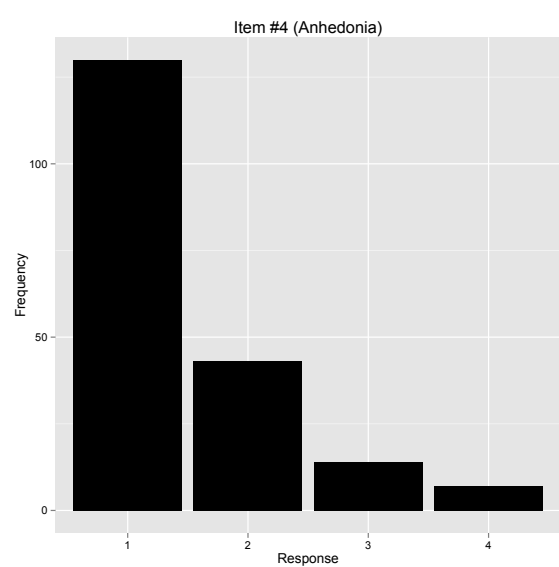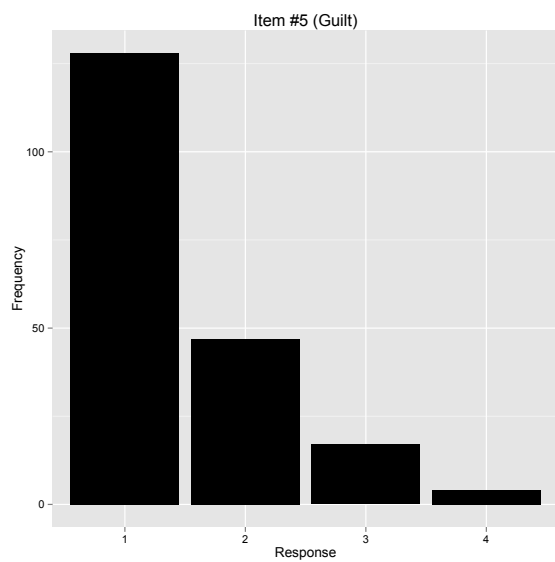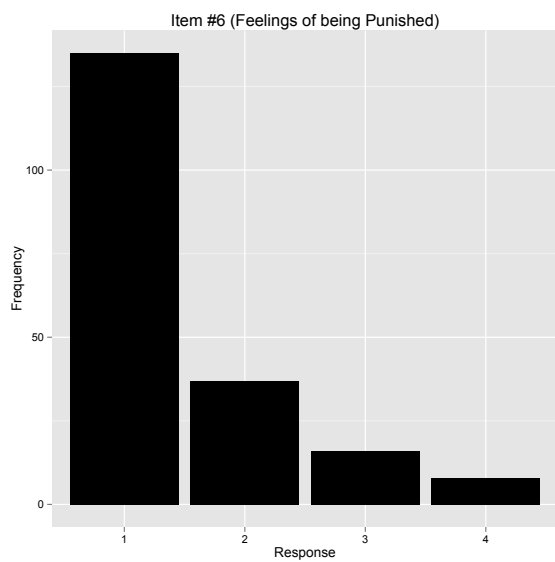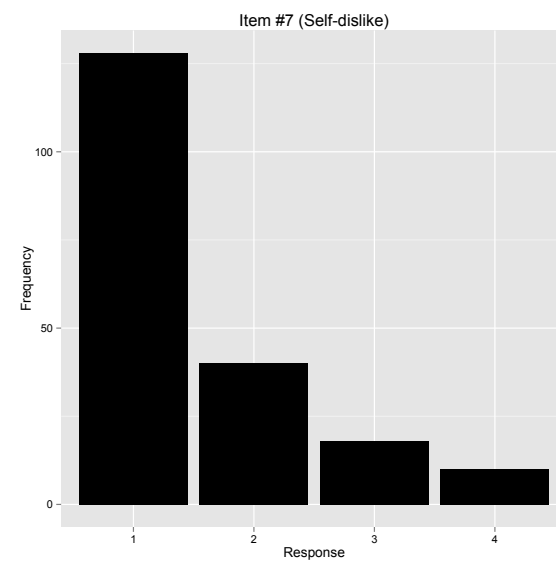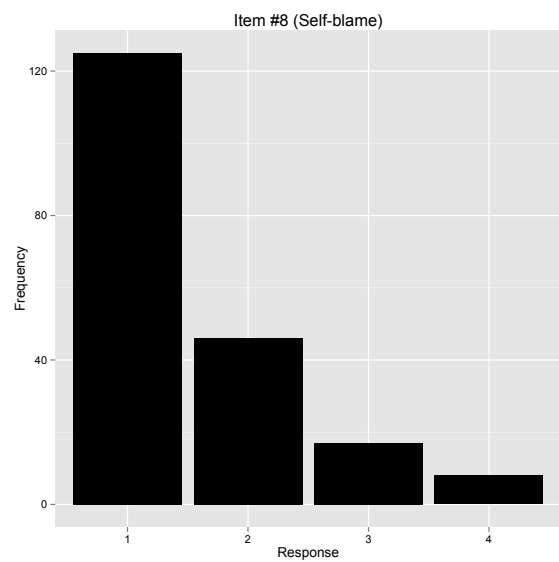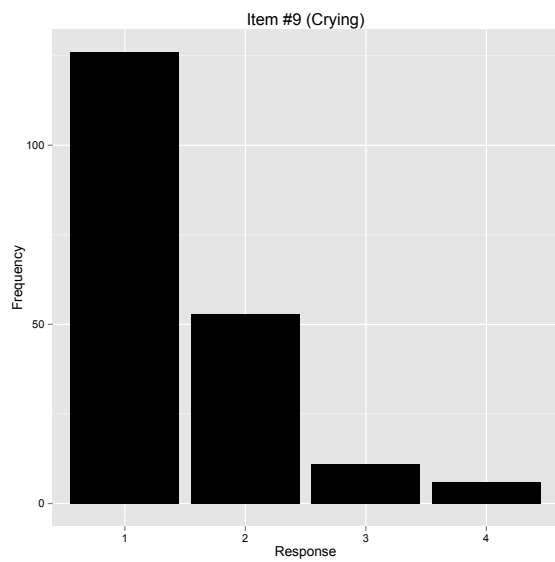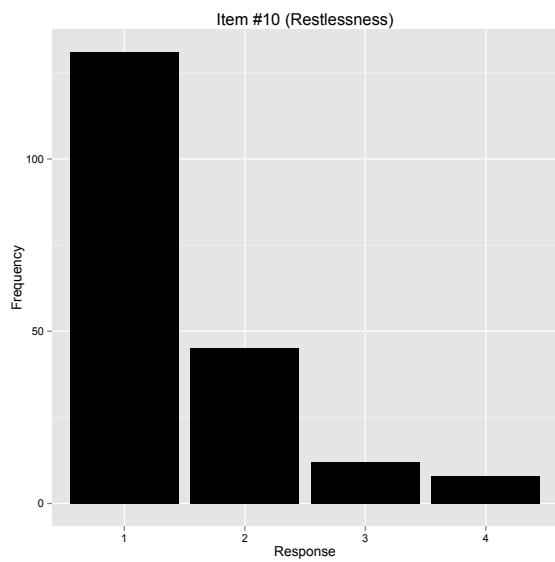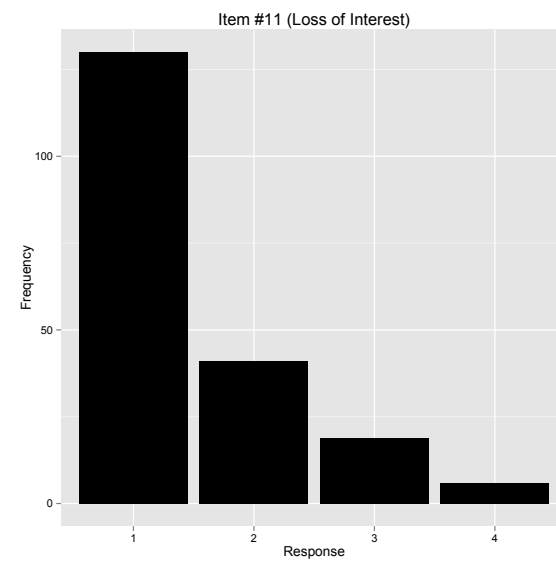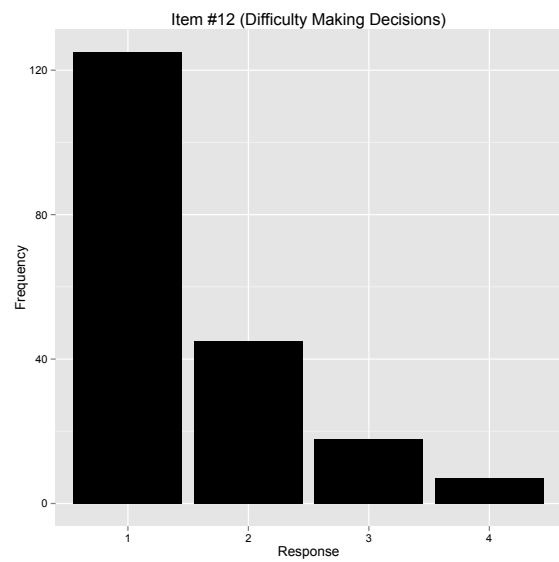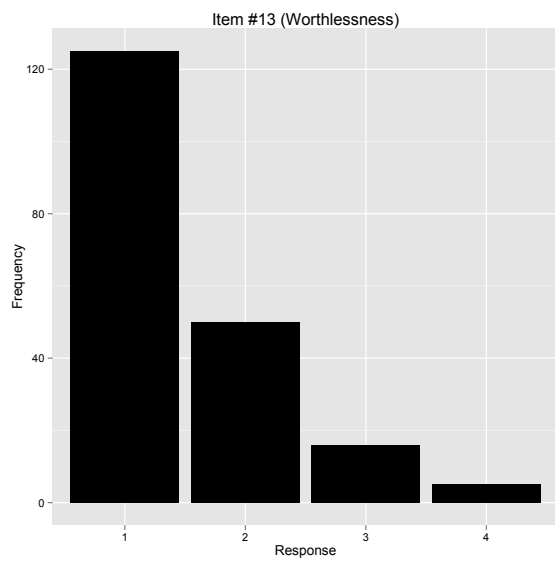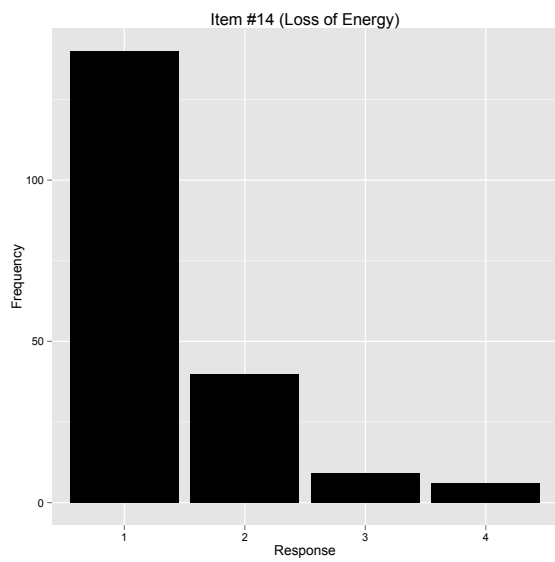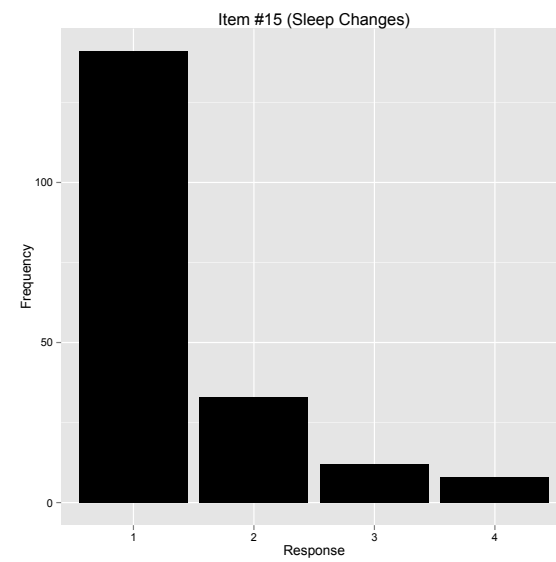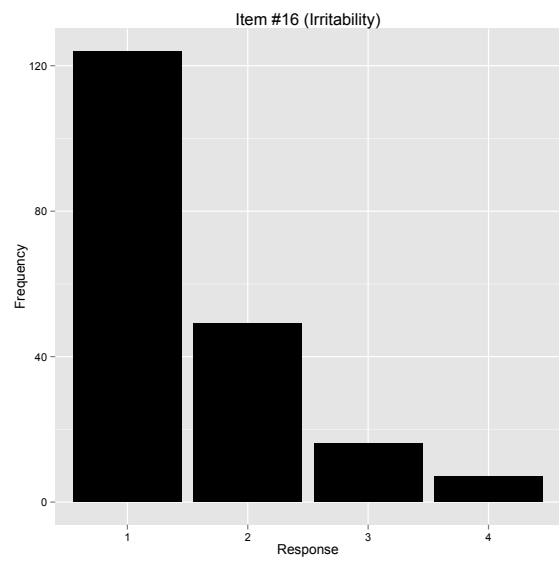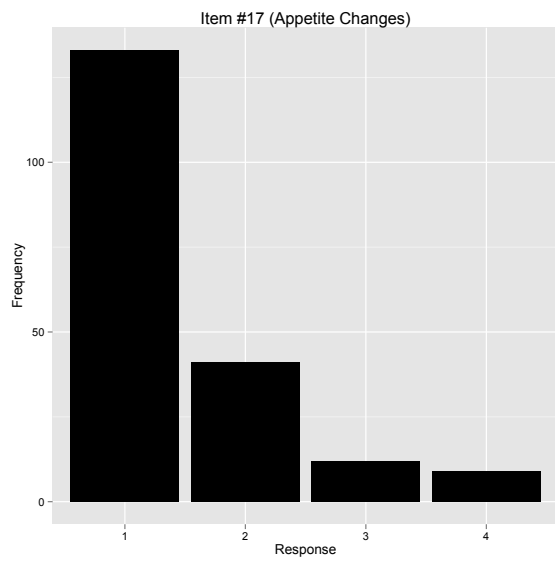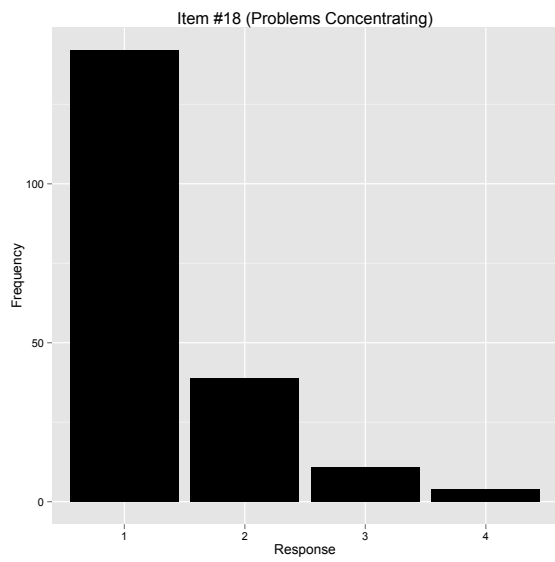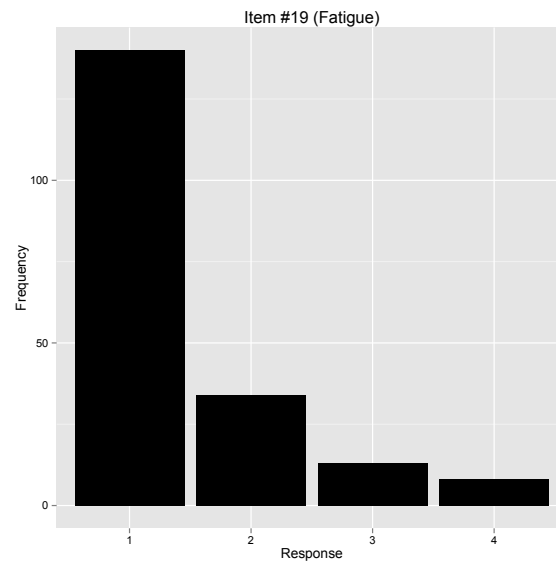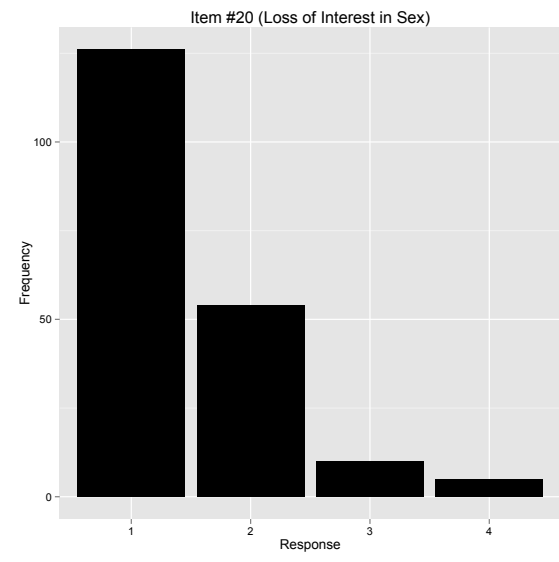

Supplement: S5 Fig — BDI-II item-level distributions for cannabis non-users (N = 196). (PDF) [file pone.0152118.s005.pdf]

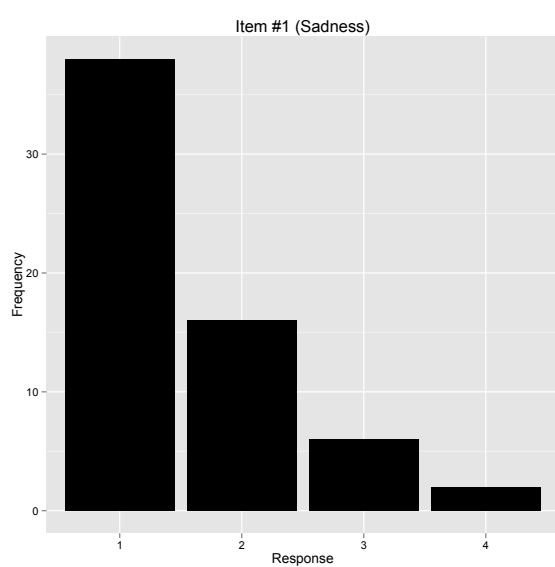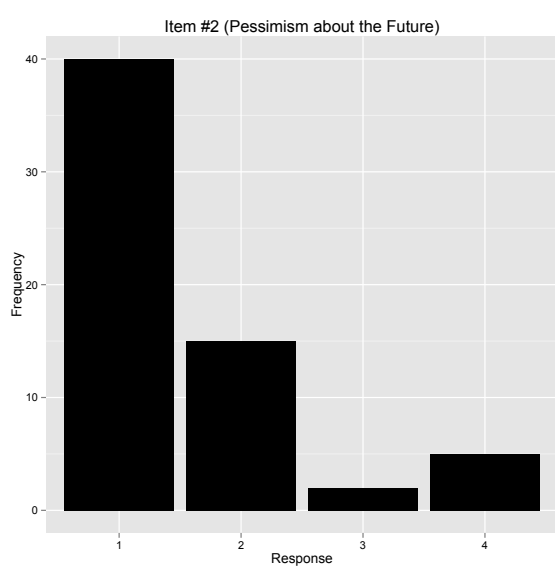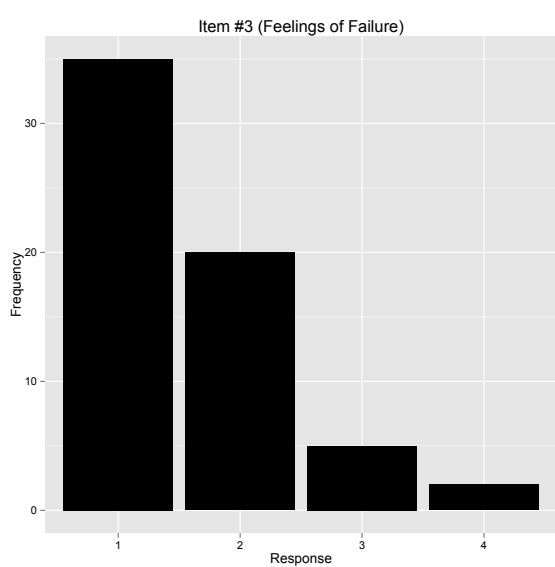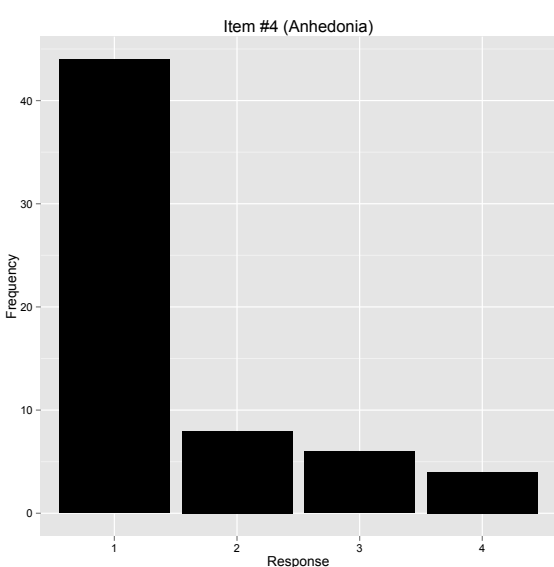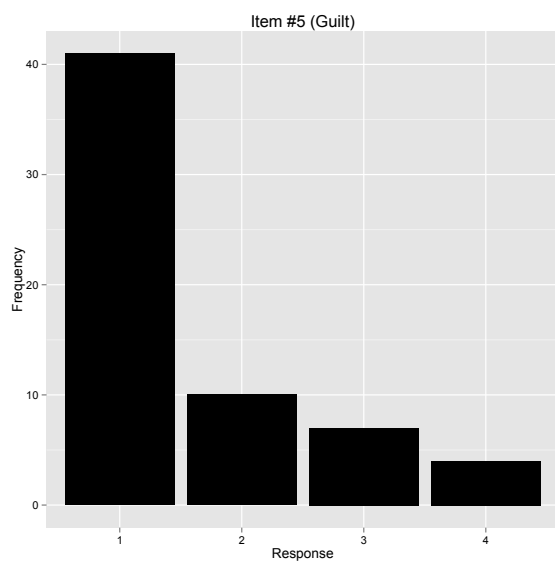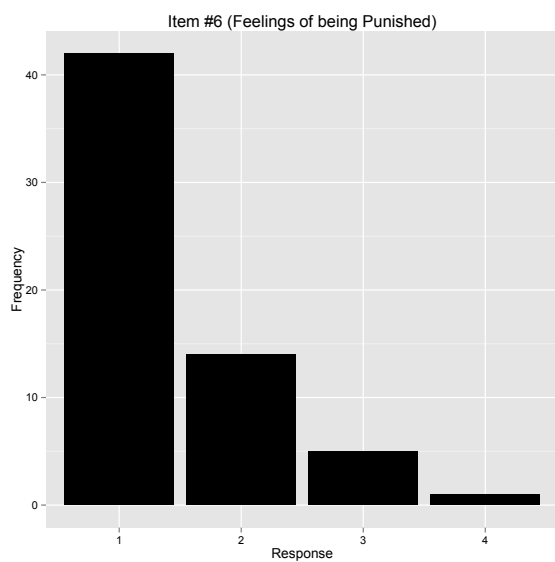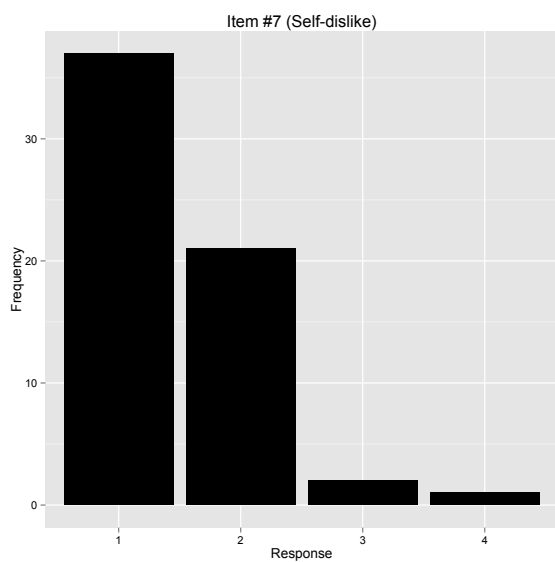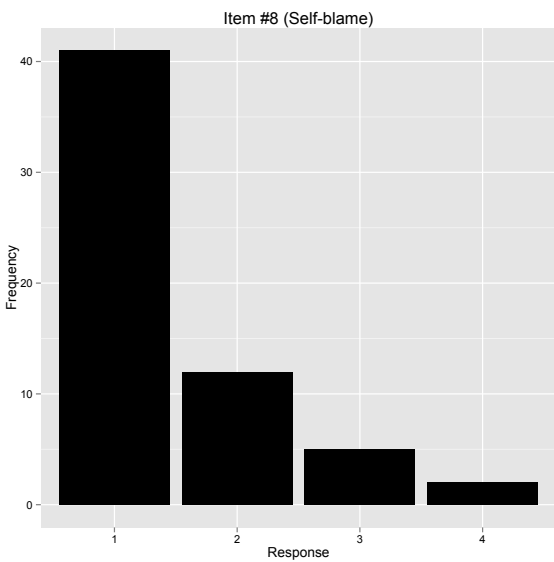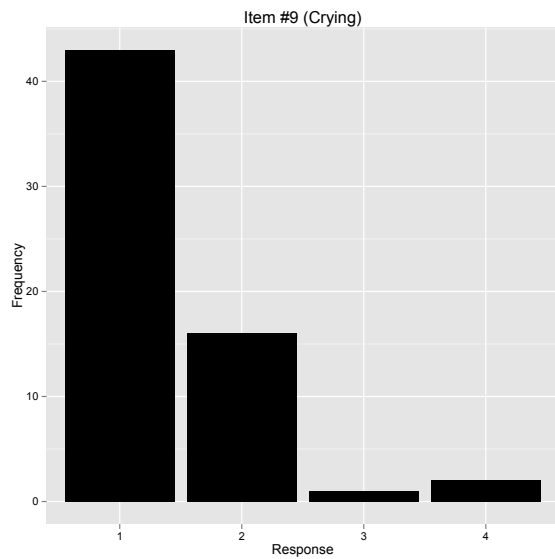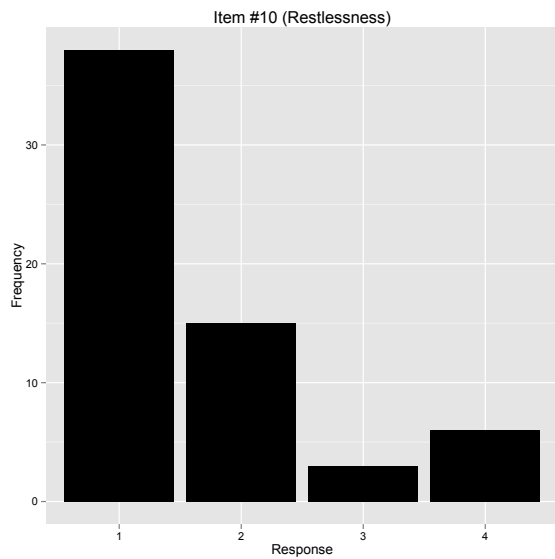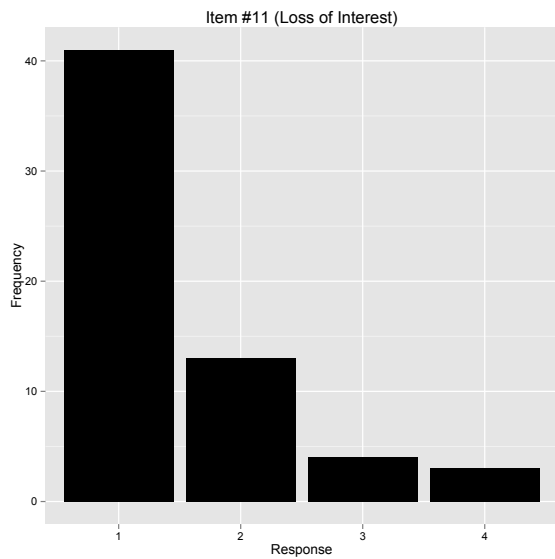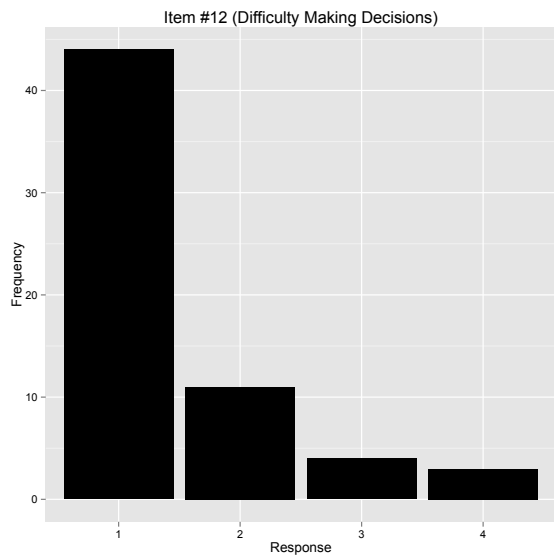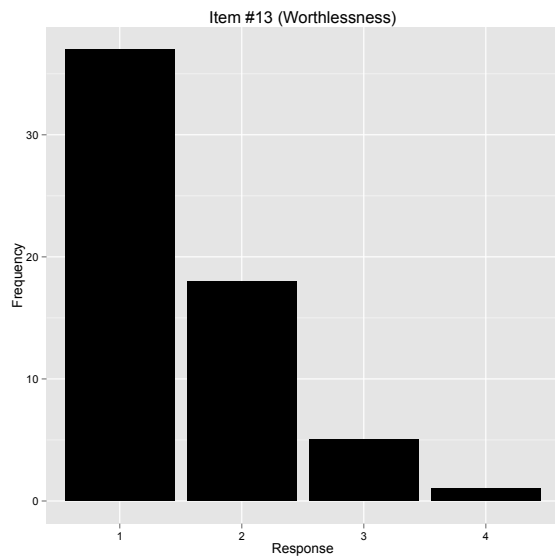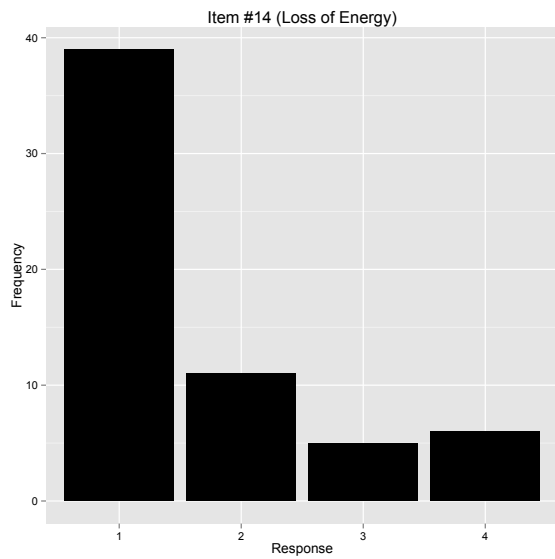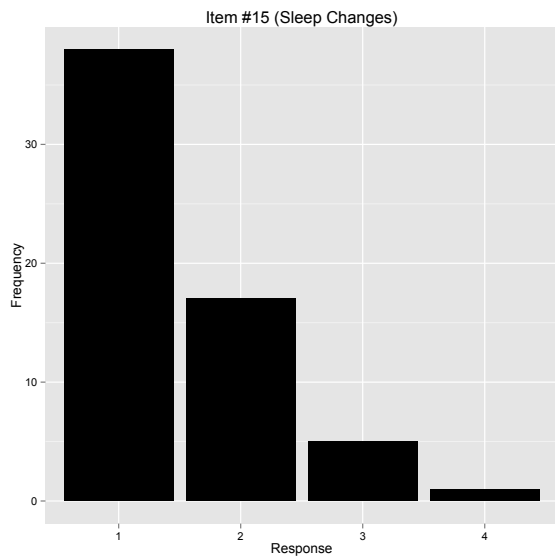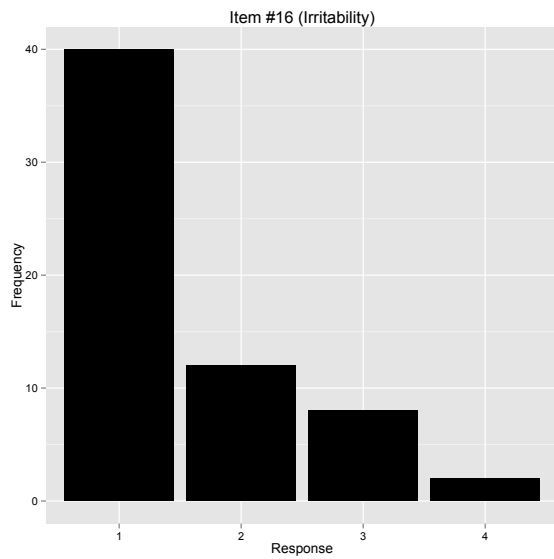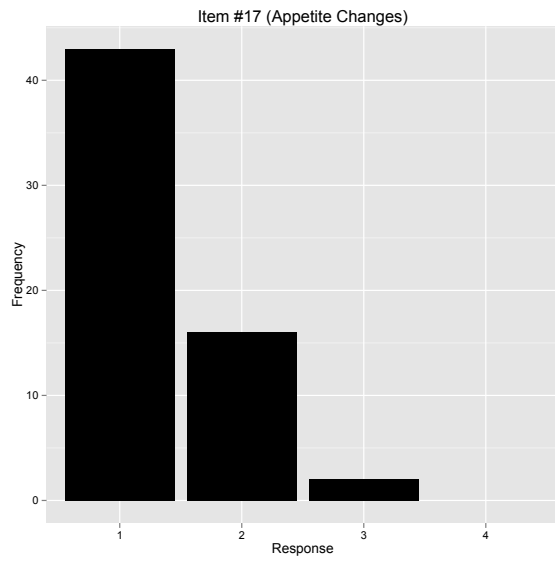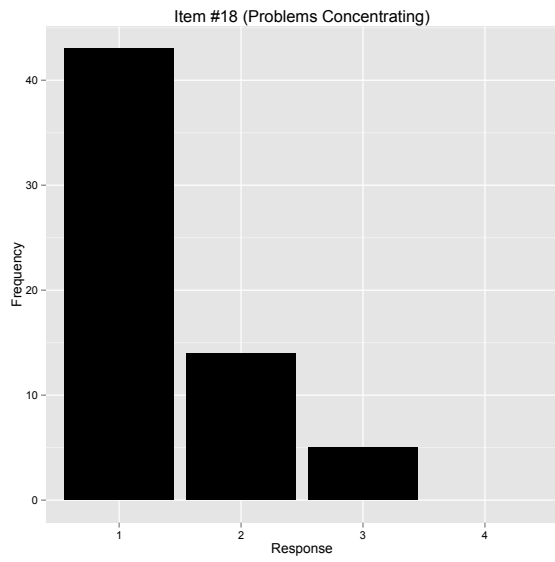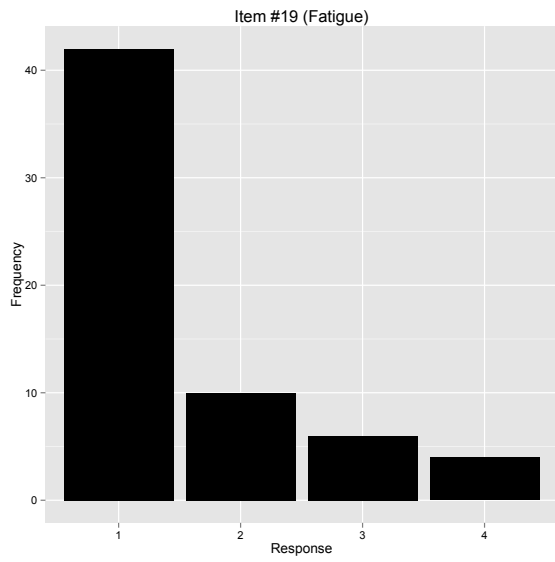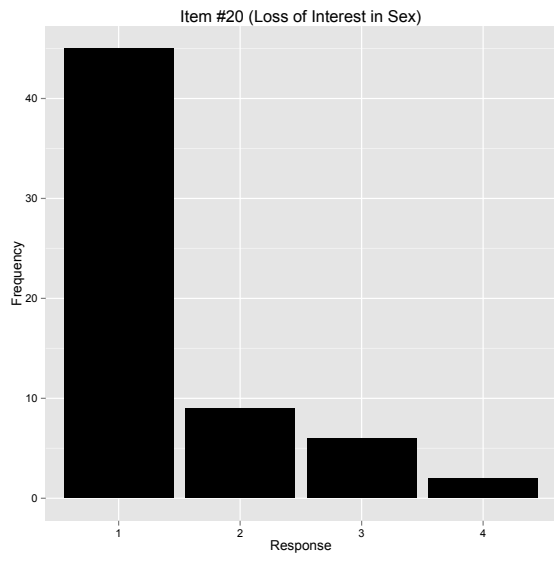

Supplement: S6 Fig — BDI-II item-level distributions for cannabis frequent-users (N = 62). (PDF) [file pone.0152118.s006.pdf]

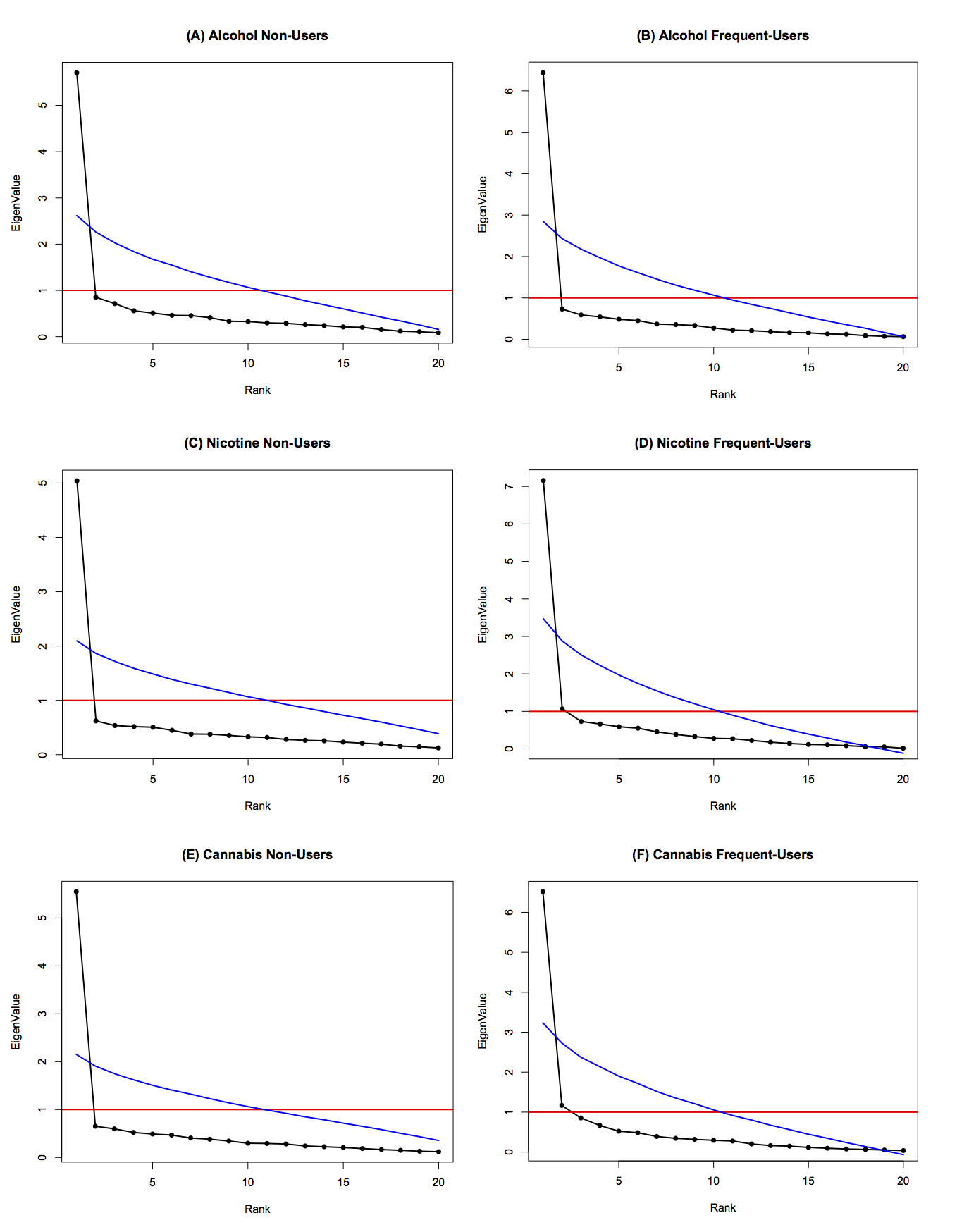

Supplement: S7 Fig — Results of exploratory factor analysis for each substance use group. Scree plots are displayed plotting the eigenvalues extracted for each group: A) alcohol non-users, B) alcohol frequent-users, C) nicotine non-users, D) nicotine frequent-users, E) cannabis non-users, F) cannabis frequent-users. Kaiser rule threshold (eigenvalue > 1) is displayed in red, and results of 1,000 parallel analyses displayed in blue. Eigenvalues above the parallel analysis threshold were retained. (TIFF) [file pone.0152118.s007.tiff]
