# Supplementary material for: Substance Use and Depression Symptomatology: Measurement Invariance of the Beck Depression Inventory (BDI-II) among Non-Users and Frequent-Users of Alcohol, Nicotine and Cannabis
Source: PLoS One. 2016 Apr 5;11(4):e0152118. doi: 10.1371/journal.pone.0152118 (PMC4821457; doi:10.1371/journal.pone.0152118)
Supplement: S1 Table — (DOCX) [file pone.0152118.s008.docx]

S1 Table. BDI-II Item Response Options

| **Item** | **Response Option** | | | | | | |
| --- | --- | --- | --- | --- | --- | --- | --- |
|  | **1** | **2** | **3** | **4** | **5** | **6** | **7** |
| Item #1 | I do not feel sad | I feel sad much of the time | I am sad all of the time | I am so sad or unhappy that I can't stand it |  |  |  |
| Item #2 | I am not discouraged about my future | I feel more discouraged about my future than I used to be | I do not expect things to work out for me | I feel my future is hopeless and will only get worse |  |  |  |
| Item #3 | I do not feel like a failure | I have failed more than I should have | As I look back, I see a lot of failures | I feel I am a total failure |  |  |  |
| Item #4 | I get as much pleasure as I ever did from the things I enjoy | I don't enjoy things as much as I used to | I get very little pleasure from the things I used to enjoy | I can't get pleasure from the things I used to enjoy |  |  |  |
| Item #5 | I don't feel particularly guilty | I feel guilty over many things I have done or should have done | I feel quite guilty most of the time | I feel guilty all of the time |  |  |  |
| Item #6 | I don't feel I am being punished | I feel I may be punished | I expect to be punished | I feel I am being punished |  |  |  |
| Item #7 | I feel the same about myself as ever | I have lost confidence in myself | I am disappointed in myself | I dislike myself |  |  |  |
| Item #8 | I don't criticize or blame myself more than usual | I am more critical of myself than I used to be | I criticize myself for all of my faults | I blame myself for everything bad that happens |  |  |  |
| Item #9 | I don't cry anymore than I used to | I cry more than I used to | I cry over every little thing | I feel like crying, but I can't |  |  |  |
| Item #10 | I am no more restless or wound up than usual | I feel more restless or wound up than usual | I am so restless or agitated that it's hard to stay still | I am so restless or agitated that I have to keep moving or doing something |  |  |  |

S1 Table (continued). BDI-II Item Response Options

| **Item** | **Response Option** | | | | | | |
| --- | --- | --- | --- | --- | --- | --- | --- |
|  | **1** | **2** | **3** | **4** | **5** | **6** | **7** |
| Item #11 | I have not lost interest in other people or activities | I am less interested in other people or things than I used to be | I have lost most of my interest in other people or things | It's hard to get interested in anything |  |  |  |
| Item #12 | I make decisions about as well as ever | I find it more difficult to make decisions than usual | I have much greater difficulty in making decisions than I used to | I have trouble making any decisions |  |  |  |
| Item #13 | I do not feel I am worthless | I don't consider myself as worthwhile as I used to | I feel more worthless as compared to other people | I feel utterly worthless |  |  |  |
| Item #14 | I have as much energy as ever | I have less energy than I used to have | I don't have enough energy to do very much | I don't have enough energy to do anything |  |  |  |
| Item #15 | I wake up 1-2 hours early and can't get back to sleep | I sleep a lot less than usual | I sleep somewhat less than usual | I have not experienced any change in my sleeping patterns | I sleep somewhat more than usual | I sleep a lot more than usual | I sleep most of the day |
| Item #16 | I am no more irritable than usual | I am more irritable than usual | I am much more irritable than usual | I am irritable all the time |  |  |  |
| Item #17 | I have no appetite at all | My appetite is much less than before | My appetite is somewhat less than usual | I have not experienced any change in my appetite | My appetite is somewhat more than usual | My appetite is much greater than usual | I crave food all the time |
| Item #18 | I can concentrate as well as ever | I can't concentrate as well as usual | It's hard to keep my mind on anything for very long | I find I can't concentrate on anything |  |  |  |
| Item #19 | I am no more tired or fatigued than usual | I get more tired or fatigued more easily than usual | I am too tired or fatigued to do a lot of the things I used to do | I am too tired or fatigued to do most of the things I used to do |  |  |  |

S1 Table (continued). BDI-II Item Response Options

| **Item** | **Response Option** | | | | | | |
| --- | --- | --- | --- | --- | --- | --- | --- |
|  | **1** | **2** | **3** | **4** | **5** | **6** | **7** |
| Item #20 | I have not noticed any recent change in my interest in sex | I am less interested in sex than I used to be | I am much less interested in sex now | I have lost interest in sex completely |  |  |  |
